# Vitamin D levels and deficiency with different occupations: a systematic review

**DOI:** 10.1186/s12889-017-4436-z

**Published:** 2017-06-22

**Authors:** Daniel Sowah, Xiangning Fan, Liz Dennett, Reidar Hagtvedt, Sebastian Straube

**Affiliations:** 1 Division of Preventive Medicine, Department of Medicine, University of Alberta, 5-30 University Terrace, 8303-112 Street, Edmonton, AB T6G 2T4 Canada; 2 JW Scott Health Sciences Library, University of Alberta, Edmonton, Canada; 3 Department of Accounting, Operations and Information Systems, School of Business, University of Alberta, Edmonton, Canada

**Keywords:** Vitamin D level, vitamin D deficiency, 25-hydroxyvitamin D (25-(OH)D), occupation, systematic review

## Abstract

**Background:**

Vitamin D deficiency is prevalent worldwide, but some groups are at greater risk. We aim to evaluate vitamin D levels in different occupations and identify groups vulnerable to vitamin D deficiency.

**Methods:**

An electronic search conducted in Medline, Embase, the Cochrane Central Register of Controlled Trials, and CINAHL Plus with Full Text generated 2505 hits; 71 peer-reviewed articles fulfilled the inclusion criteria. Occupations investigated included outdoor and indoor workers, shiftworkers, lead/smelter workers, coalminers, and healthcare professionals. We calculated the pooled average metabolite level as mean ± SD; deficiency/insufficiency status was described as % of the total number of subjects in a given category.

**Results:**

Compared to outdoor workers, indoor workers had lower 25-hydroxyvitamin D (25-(OH)D) levels (40.6 ± 13.3 vs. 66.7 ± 16.7 nmol/L; *p* < 0.0001). Mean 25-(OH)D levels (in nmol/L) in shiftworkers, lead/smelter workers and coalminers were 33.8 ± 10.0, 77.8 ± 5.4 and 56.6 ± 28.4, respectively. Vitamin D deficiency (25-(OH)D < 50 nmol/L), was high in shiftworkers (80%) and indoor workers (78%) compared to outdoor workers (48%). Among healthcare professionals, medical residents and healthcare students had the lowest levels of mean 25-(OH)D, 44.0 ± 8.3 nmol/L and 45.2 ± 5.5 nmol/L, respectively. The mean 25-(OH)D level of practising physicians, 55.0 ± 5.8 nmol/L, was significantly different from both medical residents (*p* < 0.0001) and healthcare students (*p* < 0.0001). Nurses and other healthcare employees had 25-(OH)D levels of 63.4 ± 4.2 nmol/L and 63.0 ± 11.0 nmol/L, respectively, which differed significantly compared to practising physicians (*p* = 0.01), medical residents (*p* < 0.0001) and healthcare students (*p* < 0.0001).

Rates of vitamin D deficiency among healthcare professionals were: healthcare students 72%, medical residents 65%, practising physicians 46%, other healthcare employees 44%, and nurses 43%. Combined rates of vitamin D deficiency or insufficiency (25-(OH)D < 75 nmol/L) were very high in all investigated groups.

Potential confounders such as gender and body composition were not consistently reported in the primary studies and were therefore not analyzed. Furthermore, the descriptions of occupational characteristics may be incomplete. These are limitations of our systematic review.

**Conclusions:**

Our review demonstrates that shiftworkers, healthcare workers and indoor workers are at high risk to develop vitamin D deficiency, which may reflect key lifestyle differences (e.g. sunlight exposure). This may help target health promotion and preventive efforts.

**Electronic supplementary material:**

The online version of this article (doi:10.1186/s12889-017-4436-z) contains supplementary material, which is available to authorized users.

## Background

Although there has been recent improvement in vitamin D status in the North American population, the prevalence of insufficiency remains high. About 70%–97% of Canadians are estimated to demonstrate vitamin D insufficiency [1] and approximately 40% of the US population are estimated to be vitamin D deficient [2]. While vitamin D has traditionally been shown to be involved in calcium homeostasis [1] and bone health [2], recent evidence suggests several roles not limited to the musculoskeletal system. An inadequate level of vitamin D has been linked to a number of diseases including metabolic disorders, autoimmune conditions, psychiatric, respiratory and cardiovascular disorders, and cancers as well as osteoporosis and osteomalacia [2–4]. The widespread systemic effects of vitamin D have been attributed to the ubiquitous expression of vitamin D receptors in various organ systems [2, 5].

Vitamin D is synthesized in vivo when solar ultraviolet B (UVB) radiation interacts with the precursor molecule, 7-dehydrocholesterol, in the skin [2, 6, 7]. Another important source of vitamin D is dietary intake and supplementation, although endogenous production is estimated to account for 90% of total vitamin D in healthy individuals, and any activity that reduces sunlight exposure will tend to reduce vitamin D levels [2, 8]. Whether from endogenous production or dietary sources, vitamin D is subsequently transported in the blood (bound to vitamin D-binding protein) to the liver where it is hydroxylated to 25-hydroxyvitamin D (25-(OH)D) [7]. 25-(OH)D is further converted to the metabolically active form, 1α, 25-dihydroxyvitamin D (1α, 25-(OH)_2_D), primarily in the kidneys [9]. In the present study, the term ‘vitamin D’ was used in the context of status, i.e., deficiency, insufficiency or sufficiency, while 25-(OH)D referred to serum levels of the metabolite.

Because of its half-life in blood of ~2–5 weeks, the circulating concentration of 25-(OH)D has been used as a measure of vitamin D status in individuals [6]. Serum 25-(OH)D concentrations lower than 50 nmol/L (20 ng/mL) appear to be detrimental to bone health [10]; however, optimal serum 25-(OH)D concentrations have not been established with respect to other outcomes, and there is lack of agreement on how deficiency should be defined, or how best to conduct population-based screening for vitamin D deficiency [11]. Despite a lack of consensus on optimal levels, it is becoming abundantly evident that vitamin D deficiency and its associated untoward health outcomes are a worldwide phenomenon [12, 13].

As vitamin D synthesis is highly dependent on sunlight, factors and conditions associated with decreased time spent outdoors can be expected to adversely impact vitamin D status. Shiftwork represents work that occurs outside the traditional 9 a.m. to 5 p.m. workday and may include evening or night shift work, with or without rotating shifts. An estimated 28% of working Canadians [14], 17% of Americans [15], and 22% of European workers [16] work outside the traditional 9 a.m. to 5 p.m., Monday through Friday schedule. Shiftwork has been epidemiologically associated with a number of health conditions, including sleep disturbances, cardiovascular disorders, gastrointestinal and digestive problems, and increased cancer risk, among others [17–19]. Little is known about the role of vitamin D deficiency with respect to the adverse health outcomes related to shiftwork, although vitamin D deficiency has been previously postulated as a mechanism of shiftwork-related cancers [20]; shiftworkers can plausibly be expected to have lower serum vitamin D levels due to reduced exposure to sunlight or altered dietary intake of vitamin D-rich foods. Additionally, other occupational groups (e.g. indoor workers) may be at risk of vitamin D deficiency through this same mechanism of reduced sunlight exposure, and it has been previously noted that there has been comparatively little research into the health of indoor nonindustrial workers [21].

Understanding the levels of vitamin D and prevalence of vitamin D deficiency in various occupational categories can inform public health attempts to reduce vitamin D deficiency and ensure improved population health outcomes. The link between vitamin D levels and occupation has previously been explored in the published literature, and low levels of vitamin D have been demonstrated in some occupational groups with expected low exposure to sunlight. For example, a recent study in bakers concluded that vitamin D insufficiency was very common, especially in night workers [22]. A large study from Korea found that the risk of vitamin D deficiency was significantly increased for shift work and office work [23]. An Indonesian study likewise showed that vitamin D deficiency may occur in women with indoor occupations [24]. In contrast, other evidence confirms that outdoor workers have comparatively high serum vitamin D levels [25–27]. However, the association between occupational factors (e.g. shiftwork, indoor work, work activities) and vitamin D levels is far from clear in the literature, and vitamin D deficiency in working populations does not seem to be entirely explained by sunlight exposure. A Japanese study on shiftworkers comparing serum 25-(OH)D levels in fixed daytime workers to rotating workers with or without night shift demonstrated no significant differences [28]. Additionally, vitamin D deficiency can be prevalent among certain groups of workers, for example health care workers, even in regions with high sunshine exposure [29].

To our knowledge, there has been no previous systematic attempt to examine the effect of occupation on vitamin D status, or determine the prevalence of vitamin D deficiency in different occupational groups, despite the importance of work in most workers’ lives. Additionally, we are unaware of any current guidelines on screening for vitamin D deficiency or vitamin D supplementation, which include specific guidance for workers, or risk stratification elements based on occupational factors. The present article aims to provide evidence to address these gaps.

## Methods

The objective of the present study is to investigate serum vitamin D levels, and prevalence of insufficiency and deficiency in different occupational categories to identify groups of workers at particular risk of vitamin D deficiency or insufficiency.

### Study eligibility criteria

We sought observational studies describing measured vitamin D levels or prevalence of vitamin D deficiency or insufficiency in a working population. We included studies where a distinct group of workers was compared with one or more groups of other workers or non-working individuals, and studies on students provided they were students of a specific vocation (e.g. healthcare students). Otherwise, we excluded studies on students. We excluded studies performed on subjects in the military, professional athletes, and astronauts, and studies taking place in Antarctica, because they were deemed less relevant to our study objective. We also excluded trials of vitamin D supplementation in working populations. Only peer-reviewed full journal articles were included; we excluded review articles, abstracts and conference proceedings, as well as articles not published in English or German. We included papers in which data were derived from large population based cohort studies in a defined occupational group (e.g. the Nurses’ Health Study, Physicians’ Health Study, etc.). For case-control studies, we excluded information on cases but included information on controls, as they would arguably have been representative of the study population and were not defined by a disease state.

### Search strategy

Electronic database searches were conducted by a health sciences librarian (LD) in July 2015 and updated in March 2016 in Medline (including in process and other non-indexed citations as well as Medline Daily), Embase, the Cochrane Central Register of Controlled Trials, and CINAHL Plus with Full Text. The searches used an extensive combination of keywords and subject headings for the concepts of vitamin D and occupation to identify relevant studies. Studies that only included participants who were 18 and under or 65 and older were excluded as they were assumed not to be about working populations. Furthermore, studies where the described occupations could not be meaningfully grouped into occupational categories have been excluded from this review. The full version of the electronic search strategies can be found in ‘Additional file 1: Search strategies’. Reference lists of retrieved articles and reviews in the field were assessed to identify additional publications of relevance. Search results and full-text articles were screened independently by two investigators (XF, DS). Wherever there was a disagreement in the selection of relevant articles between the two investigators, the senior investigator (SS) made the final judgement based on the established inclusion/exclusion criteria.

### Assessment of study quality and data extraction

We extracted data on the study ID, number of subjects, location/latitude, measured vitamin D levels (25-hydroxyvitamin D, 25-hydroxyvitamin D2, 25-hydroxyvitamin D3, and 1α, 25-dihydroxyvitamin D) and on the prevalence of vitamin D deficiency and insufficiency. Additionally, we extracted data on season of the year and the assay type from articles where this information was available. Where the latitude of the location of study was not provided but the name of the city or country of study was given, it was obtained from an online tool on the National Aeronautics and Space Administration (NASA) website by entering the city or country name and searching for the respective latitude [30]. The extracted data also included first author, year of publication, study type and occupational group. Data extraction was performed by one investigator and independently verified by a second (XF, DS). Discrepancy between the two investigators was resolved by consulting with the senior investigator (SS).

The quality of the included studies was assessed based on previously employed criteria [12], which included the representativeness (selection of study subjects) of the individual study report, the validity of method used to measure vitamin D levels [31, 32], and assay reliability, which pertains to the intra- and inter-assay coefficients of variation of the assay. However, in the present report, representativeness of study participants was not a major concern since serum 25-(OH)D status was assessed in workers of a given occupational category; therefore, we did not include this factor in our evaluation of study quality.

One criterion that we used to assess study quality was whether the authors mentioned the season of the year in which the study was conducted, as there is a seasonal variation in vitamin D levels [4] which is an important confounder when interpreting vitamin D levels. Additionally, a particular study was considered valid if the assay technique to determine vitamin D levels was in keeping with the International Vitamin D Quality Assessment Scheme [33]. Finally, to be considered reliable, the inter-assay coefficient of variation (CV) must be less than 15%, while the intra-assay CV must be below 10% [12, 33]. A study was considered to be of high quality if all three criteria (reporting on seasonality, validity and reliability) were met; of medium quality when only two of the criteria were satisfied; when only one criterion was met, we considered such a study to be of low quality; and when none of our quality criteria were met, the study was considered to be of very low quality.

### Data synthesis

The mean levels of measured vitamin D metabolites (25-(OH)D and/or 1α, 25-(OH)_2_D) were extracted from included studies for each occupational category. Studies where metabolite levels were provided as mean ± SD/SE (standard deviation/standard error) were included in further analysis to compute the overall mean for the particular occupational group. To enable pooling of data from different studies for a particular occupational group, we also standardized the measure of spread by converting SE to SD, where applicable, using the formula, *SD* = *SE x* √ *N*, where N is the sample size or number of subjects.

Following conversion to SD, the pooled SD was calculated by combining the individually weighted SD based on the formula below [34]:

1 $$ {\mathrm{S}}_{\mathrm{P}}^2=\frac{\left({\mathrm{n}}_1-1\right){\mathrm{S}\mathrm{D}}_1^2+\left({\mathrm{n}}_2-1\right){\mathrm{S}\mathrm{D}}_2^2}{{\mathrm{n}}_1+{\mathrm{n}}_2-2} $$

S_p_^2^_=_ pooled variance.

n_1_ = sample size of group 1.

n_2_ = sample size of group 2.

SD_1_ = standard deviation of group 1.

SD_2_ = standard deviation of group 2.

Pooled standard error, SEp, was calculated according to the following formula [35]:

2 $$ SEp= Sp\sqrt{\frac{1}{{\mathrm{n}}_1}+\frac{1}{{\mathrm{n}}_2}} $$

When averages were presented in the study as median ± interquartile ranges (IQRs), the values were converted into estimated mean ± SD based on a previously established approach [36–38]. Data were not included in the final meta-analysis if only the median values were provided in the absence of IQRs [39, 40] or geometric means reported without indication of a measure of spread [41, 42]. Whenever there were three or more studies from an occupational group, which reported data as mean ± SD, a meta-analysis was conducted on those studies by pooling or combining the means and SDs using the method for combining means and SDs as described in the Cochrane Handbook for Systematic Reviews of Interventions [43]. Based on this approach, the overall mean of each occupational category was calculated by averaging the means of individual studies weighted by the number of subjects of each study.

All units of measurement of vitamin D concentration were standardized to the S.I. units, nmol/L for 25-(OH)D or pmol/L for 1α, 25-(OH)_2_D, by multiplying the imperial unit (ng/ml or pg/ml, respectively) by a factor of 2.5 or 2.4, respectively [12]. The number of studies reporting on levels of 1α, 25-(OH)_2_D were too few to permit quantitative comparison of results obtained between different occupational groups. Results were therefore mainly analyzed and compared relative to the average serum levels of 25-(OH)D computed from each occupational category. To evaluate the differences in the prevalence of vitamin D insufficiency and deficiency between different occupational groups, the proportion of study subjects who were either deficient or insufficient in the relevant groups were compared.

Due to the lack of consensus regarding the level of 25-(OH)D that constitutes vitamin D deficiency, we adopted the widely employed Endocrine Society’s (ES) cut-offs as standard definition to compare the degree of deficiency between different occupational groups. The ES has defined vitamin deficiency as a serum 25-(OH)D concentration of <20 ng/ml (<50 nmol/L), a serum level between 20 ng/ml (50 nmol/L) and 30 ng/ml (<75 nmol/L) as insufficiency, and a level > 30 ng/ml (>75 nmol/L) as adequate to maintain normal physiological function [2, 44]. However, when levels of deficiency were provided in the absence of mean 25-(OH)D level, such studies were not included in further meta-analysis.

We chose the weighted average of the proportions of insufficient and deficient vitamin D status as the baseline for comparison with specific occupational groups. We calculated relative risk (RR) by finding the percentage increase (or decrease) in proportion, compared to this baseline with a 95% confidence interval (CI).

### Statistical analysis

Data on the average serum 25-(OH)D levels of each occupational group are reported as mean ± SD. The levels of 25-(OH)D deficiency or combined deficiency/insufficiency of each occupational group are reported as a percentage of the total number of subjects in the given group. Whether the difference in means between occupational categories was statistically significant, was determined with the unpaired Student’s t-test. We used a Chi-squared test to determine the significance of differences between proportions of vitamin D deficiency or deficiency/insufficiency between occupational groups. A *p*-value of <0.05 was considered statistically significant. The Bonferroni procedure was employed to test whether pairwise differences were statistically significant, while retaining an overall level of significance of 5%. Data were extracted and analyzed in Microsoft Excel and *p*-values were estimated with GraphPad software.

## Results

The electronic database search generated 1991 records after deduplication, of which 87 primary studies (all in English) were considered potentially relevant, based on title and abstract screening, and available as full text journal articles. An additional 17 articles were identified by searching reference lists of previously identified articles or reviews. Of this total of 104 articles, 33 were ultimately excluded after review of the full-text article, leaving 71 articles to be included for further quantitative analysis as shown in ‘Additional file 2: Study selection’. The total number of subjects for all included studies was at least 53,345 (one study did not report on the number of subjects) and the sample sizes ranged from 4 to 10,646 subjects per study as shown in Table 1. The overall proportion of participants who were women was 65%. Publication dates ranged from 1971 to 2016. Based on the latitudes of study locations, the included studies spanned a range of latitudes from 3° N (Indonesia) to 64° N (Reykjavik, Iceland) in the Northern hemisphere, and 23° S (Sao Paulo, Brazil) to 30° S (Porto Alegre, Brazil) in the Southern hemisphere (Table 1 and Additional file 3: Figure S4A).

**Table 1:** Characteristics of included studies

Group	Author (year)	Occupational Detail	Number of Subjects	Location/Latitude	Study type	25-(OH)D		Notes
						Mean ± SD (nmol/L)	% Deficiency	
Outdoor Workers								
	Haddad and Chyu (1971) [102]	Lifeguards	8	St. Louis, Missouri, USA (38°63′ N)	Descriptive	160.7 ± 21.7		
	Devgun (1981) [85]	Gardeners	18	Dundee/Scotland (56°30′N)	Descriptive	59.4 ± 19.7		
	Devgun (1981) [25]	Male outdoor workers	20	Scotland (56°30′N)	Descriptive	62.1 ± 18.8		
	Devgun (1983) [103]	1.Outdoor workers	9	Scotland (56°30′N)	Descriptive	73 (1977), 54 (1979)		Average reported as median
		2. Outdoor workers	9			55 (1976), 84 (1977)		Excluded from meta-analysis
	Azizi (2009) [104]	Agriculture, physical education, construction	122	Beer Sheva, Israel (31°25′ N)	Case-control	67.6 ± 21.3		
	Norsang (2009) [105]	1. Farmers	20	Shigatse, China (29°19′ N)	Descriptive	81.0 ± 18.0	<75 nmol/L: 40% <50 nmol/L: 0%	
		2. Farmers	6	Tingri, China (28°34′N)		67.0 ± 27.0	<75 nmol/L: 67% <50 nmol/L: 17%	
		3. Farmers	6	Chonggye, China (29°02′ N)		46.0 ± 27.0	<75 nmol/L: 83% <50 nmol/L: 17%	
	Azizi (2012) [91]	Israel National Water Company						
		1. Group 1	34	Israel (31°05 N)	Interventional	74.4 ± 15.2		
		2. Group 2	67			98.3 ± 17.2		
						80.4 ± 27.7		
		3. Group 3	53					
	Choi (2011) [106]	Agriculture, Forestry, Fishery	644	South Korea (33° - 38°N)	Cross-sectional	61.3 ± 18.5	<75 nmol/L: 76.2% <50 nmol/L: 27.8%	
	Roomi (2015) [55]	Outdoor workers	15	Lahore, Pakistan (31°55′N)	Cross-sectional	31.4 ± 3.8		
	Oh (2015) [107]	1. Craft, equipment, machine operating, and assembling workers	2812	South Korea (33° - 38°N)	Cross-sectional		<50 nmol/L: 61.6%	
		2. Skilled agricultural, forestry, and fishery workers	2572				<50 nmol/L: 38.5%	
	Bacchel (2015) [27]	Farmers	6	North-West Punjab, India (31°15′N)	Cross-sectional		<75 nmol/L: 50% <25 nmol/L: 50%	
Indoor Workers	Devgun (1981) [85]	Laboratory staff	8	Dundee, Scotland (56°30′N)	Descriptive	43.9 ± 17.0		
	Devgun (1981) [25]	Indoor workers	9	Scotland (56°30′N)	Descriptive	42.9 ± 15.3		
	Maeda (2007) [108]	Plant and office workers	28	Sao Paulo, Brazil (23° 34′S)	Descriptive	94.0 ± 32.6		
	Gulvady (2007) [109]	Senior executives	75	Mumbai, India (19°08′ N)	Descriptive		<50 nmol/L: 83%	
	Islam (2008) [110]	Garment factory workers	200	Dhaka, Bangledesh (23°81′N)	Descriptive	36.7 ± 11.2	<50 nmol/L: 86.5%	
	Azizi (2009) [104]	Industry, civil service, etc.	104	Israel, Beer Sheva (31°25′ N)	Interventional	57.5 ± 20.8		
	Itoh (2011) [28]	Indoor daytime workers	4	Kawasaki City, Japan (35°53′N)	Interrupted time series	49.7 ± 7.9		
	Vu (2011) [111]	Office workers	213	Brisbane, Australia (27°S)	Descriptive	62.2 ± 22.5	<75 nmol/L: 43.3% <50 nmol/L: 42.5%	
	Choi (2011) [106]	1. Administration, clerical work	1047		Cross-sectional	45.8 ± 16.5	<75 nmol/L: 93.4% <50 nmol/L: 87.1%	
						53.8 ± 18.5	<75 nmol/L: 87.1% <50 nmol/L: 50%	
		2. Engineering, assembling, and technical work	572	South Korea (33° - 38°N)				
	Xiang (2013) [56]	Pregnant indoor workers	311	Guiyang, China (26°65′ N)	Descriptive	36.7 ± 17.0	<75 nmol/L: 12.5% <50 nmol/L: 83.6%	
	Cinar (2014) [112]	Premenopausal female and male office workers.	118	Ankara, Turkey (39°93′N)	Prospective observational	52.8 ± 28.4	<75 nmol/L: 24.2% <50 nmol/L: 54.3%	
	Jeong (2014) [23]	Managers, experts, specialists, etc.	2659	South Korea (35°91′N)	Descriptive	40.6 ± 18.0	<50 nmol/L: 80.4%	
	Sharma (2015) [113]	Office working women	50	Udaipur, Rajasthan, India (24°59′N)	Descriptive	46.7 ± 17.4		
	Yosephin (2015) [114]	Garment factory workers	39	Indonesia (3°35′N)	Randomized control-trial	39.5 ± 12.0	<75 nmol/L: 82%	
	Roomi (2015) [55]	Indoor workers	73	Lahore, Pakistan (31°55′N)	Cross-sectional	19.0 ± 1.1		
	Kwon (2015) [54]	Manufacturing workers	1054	Busan, Gyeongnam/South Korea (35°18′N)	Cross-sectional	22.7 ± 8.1	<50 nmol/L: 97.2%	Deficiency <25 nmol/L:68.4%
								Insufficiency: <50 nmol/L: 97.2%
	Oh (2015) [107]	1. Clerks	2357	South Korea (33° - 38°N)	Cross-sectional		<50 nmol/L: 74.7%	
		2. Managers, professionals and related work	3597				<50 nmol/L: 74.8%	
	Bacchel (2015) [27]	1. Public servants	69	North-West Punjab, India (31°15′N)	Cross-sectional		<75 nmol/L: 100% <25 nmol/L: 56.5%	
		2. Professionals (businessmen) working indoors	13				<75 nmol/L: 100% <25 nmol/L: 16.7%	
Shift Workers	Ward (2011) [41]	1. Shiftworkers with constant regular work hours	4496	United Kingdom (55°39′N)	Cohort	53.0	<50 nmol/L: 81%	Average reported as geometric mean. Excluded from meta-analysis
		2. Shiftworkers with varying number of hours worked per week	6136			52.3	<50 nmol/L: 79.4%	
	Itoh (2011) [28]	1. Rotating shift workers without night shift	4	Osaka Prefecture, Japan (34.5°N)	Cross-sectional	63.1 ± 6.3		
		2. Rotating shift workers with night shift	4			64.4 ± 8.1		
	Kim (2013) [115]	Shift workers without day shift	627	South Korea (35°91′N)	Descriptive	40.0 ± 14.7		
	Jeong (2014) [23]	Shiftworkers	969	South Korea (35°91′N)	Descriptive	40.0 ± 17.7	<50 nmol/L: 80.1%	
	Kwon (2015) [54]	Manufacturing workers	872	Busan, Gyeongnam/South Korea (35°18′N)	Cross-sectional	22.2 ± 8.1	<25 nmol/L: 71% ≥25 nmol/L: 29%	
	Romano (2015) [45]	Shiftworkers	96	Northern Italy, Milan Province, Lomabardy (45° 30′N)	Cross-sectional	33.4 ± 13.2	<75 nmol/L: 24% <50 nmol/L: 66.8%	
Lead/Smelter	Greenberg (1986) [79]	Lead and cadmium exposed workers	37	Pittsburgh & Cleveland, United States (40°44′N & 41°50′N)	Descriptive	62.5 ± 24.5	5.4%	Mean 1α, 25-(OH)_2_D: 122.7 ± 36.5 pmol/L. Vitamin D deficiency was not defined.
	Mason (1990) [78]	Lead exposed workers	63	United Kingdom (55°39′N)	Cohort			Mean 1α, 25-(OH)_2_D: 90.5 ± 29.5 pmol/L
	Chalkley (1998) [39]	Smelter workers	19	England (52°36′N)	Descriptive	Mean: 71.4 Median: 71.0		1α, 25-(OH)_2_D3 Mean: 77.3 pmol/L; Median: 84.0 pmol/L. Excluded from meta-analysis
	Kristal-Boneh (1998) [77]	1. Lead-exposed factory workers (battery and recycling)	56	Israel (31°25′ N)	Cross-sectional	86.0 ± 25.2		Mean 1α, 25-(OH)_2_D: 198 ± 64.8 pmol/L
		2. Non-lead exposed workers	90			79.0 ± 20.5		Mean 1α, 25-(OH)_2_D: 165 ± 42.3 pmol/L
	Potula (2005) [116]	Smelter workers	73	Bunker Hill, Idaho, USA (43°91′N)	Descriptive			Mean 1α, 25-(OH)_2_D: 115.9 ± 38.0 pmol/L
Coal-miners	Shuster (1981) [82]	1. Underground miners	101	Newcastle Upon Tyne (54°98′N)	Cross-sectional	73.8 ± 73.4		
		2. Surface workers	19			82.3 ± 67.6		
		3. Miners not at work	6			83.5 ± 67.4		
	Shuster (1982) [81]	1. Underground miners	60	United Kingdom (55°39′N)	Cross-sectional	58.5 ± 24.3		
		2. Surface workers	28			62.6 ± 21.7		
	Sarikaya (2006) [83]	1. Underground miners	50	Zonguldak, Turkey (41°45′N)	Cross-sectional	24.5 ± 28.2		
		2. Surface workers	50			35.3 ± 29.3		
Healthcare Students	Maeda (2007) [108]	Medical students	44	Sao Paulo (23° 34′S), Brazil	Descriptive	81.5 ± 35.8		
	Gonzalez-Padilla (2011) [117]	Medical students	103	Gran Canaria, Canary Islands (27°92′N)	Descriptive	69.6 ± 31.0	<75 nmol/L: 28.6% <50 nmol/L: 32.6%	Paper reported unit as ng/dL but ng/ml was used for calculation
	Kaehler (2012) [118]	Female healthcare professional students	215	Innsbruck, Austria (47°27′N)	Cross-sectional	50.3 ± 16.6	<75 nmol/L: 33.5% <50 nmol/L: 55.8%	
	Al-Elq (2012) [119]	Medical students	198	Dammam, Saudi Arabia (26°39′N)	Cross-sectional	21.2 ± 11.9	<75 nmol/L: 4% <50 nmol/L: 96%	
	Manickam (2012) [120]	Medical students and residents	104	Chicago, IL, USA (42°N)	Descriptive	54.0 ± 28.0	<75 nmol/L: 77%	
	Zabihiyeganeh (2014) [121]	Medical students	100	Tehran, Iran (35° 69′N)	Cross-sectional	42.0 ± 11.7	<75 nmol/L: 15% <50 nmol/L: 84%	
	Milovanovic (2015) [122]	Medical, pharmacy and dental students	86	Kragujevac, Serbia (44°N)	Descriptive	33.1 ± 12.1	<50 nmol/L: 88.4%	
Medical Residents	Haney (2004) [61]	Medical residents	34	Portland, OR, USA (45°52′ N)	Interrupted series	56.4 ± 20.1	<50 nmol/L: 38%	Paper reported unit as ng/dL but ng/ml was used for calculation
	Maeda (2007) [108]	Medical residents	49	Sao Paulo, Brazil (23° 34′S)	Descriptive	67.1 ± 27.0		
	Orlandin Premaor (2014) [123]	Medical residents	73	Porto Alegre, Brazil (30°S)	Cross-sectional	44.7 ± 20.0	<50 nmol/L: 57.4%	
	Multani (2010) [76]	Medical residents	214	Mumbai, India (19°08′ N)	Cross-sectional	31.1 ± 18.6	<50 nmol/L: 87.2%	
	Singh (2011) [124]	Medical residents	80	Varanasi, India (25°N)	Cross-sectional	22.8 ± 18.2	<75 nmol/L: 11% <50 nmol/L: 89%	
	Growdon (2012) [125]	Trainee doctors (Residents)	102	Boston, MA, USA (42°36′ N)	Descriptive	67.0 ± 26.0	<75 nmol/L: 44% <50 nmol/L: 25%	
	Mendoza (2013) [126]	Medical residents	20	Mexico City, Mexico (19°43′N)	Cross-sectional	42.4 ± 13.0	<50 nmol/L: 75%	
	Ramirez-Vick (2015) [127]	Medical residents	51	San Juan, Puerto Rico (18°47′ N)	Descriptive	54.3 ± 19.4	<75 nmol/L: 45% <50 nmol/L: 43%	
Practising Physicians	Gann (1996) [128]	Male physicians	414	United States (37°09′N)	Case-control	Median: 71.1	<50 nmol/L: 6.5%	
	Goswami (2000) [129]	Physicians and nurses	19	Delhi, India (28°61′N)	Descriptive	13.0 ± 7.9		Mean 1α, 25-(OH)_2_D: 93.6 ± 29.0 pmol/L
	Kramm (2010) [130]	Physicians	28	Madison, Wisconsin (43°07′N)	Descriptive	80.0 ± 25.0	<50 nmol/L: 21%	25-(OH)D deficiency was defined as <62.5 nmol/L
	Mahdy (2010) [75]	Physicians and nurses	340	Doha, Qatar (25°29′N)	Observational	29.3	<75 nmol/L: 9.5% <50 nmol/L: 87%	SD was not provided. Excluded from meta-analysis
	Lee (2011) [131]	Male physicians	389	United States (37°09′N)	Case-control	64.0		Only control values were used for analysis
	Haliloglu (2016) [132]	Medical doctors	Unknown	Istanbul/Turkey (41°N)	Prospective observational	Winter: 42.8 ± 22.5 Summer: 58.1 ± 24.3		Total number of healthcare workers were 190. Actual number of medical doctors was not provided
	Munter (2015) [133]	Hospital- and community-based physicians	81	Jerusalem (31.4°N), Israel	Descriptive	56.2 ± 16.3	<75 nmol/L: 24.6% <50 nmol/L: 67.6%	
Nurses	Platz (2000) [134]	Nurses	326	United States (37°09′N)	Case-control	67.0 ± 25.5		Mean 1α, 25-(OH)_2_D: Controls: 77.3 ± 20.6 pmol/L
	Eliassen (2011) [135]	Nurses	1218	United States (37°09′ N)	Case-control	62.4 ± 24.0		Only control values used
	Hattapornsawan (2012) [136]	Nurses	217	Nonthaburi, Thailand (13°86′N)	Cross-sectional		<75 nmol/L: 45.6% <50 nmol/L: 49.8%	
	Wallingford (2014) [137]	Premenopausal nurses	83	Kingston, ON, Canada (44°23′N)	Cross-sectional	83.5 ± 36.2	<50 nmol/L: 11.2%	
	Wang (2014) [138]	Nurses	584	United States (37°09′N)	Case-control	61.1 ± 22.8		Only control values used
	Haliloglu (2016) [132]	Nurses	Unknown	Istanbul/Turkey (41°N)	Prospective observational	41.8 ± 16.8		Authors did not report number of subjects
	Bertrand (2016) [139]	Nurses	835	Boston/USA (42°36′N)	Case-control	68.0 ± 25.8		Only control values used
	Madani (2015) [140]	Nurses	200	Kashan/Iran (33°98′N)	Cross-sectional	42.4 ± 52.8	<75 nmol/L: 178 (89%) ≤25 nmol/L: 91 (45.5%) <25–75 nmol/L: 87 (43.5%)	25-(OH)D 25–75 nmol/L (deficiency);
								25-(OH)D ≤ 25 nmol/L (severe deficiency)
Other Healthcare Employees	Platz (2000) [59]	Health professionals	150	United States (37°09′N)	Cohort	45.5 ± 15.0		Mean 1α, 25-(OH)_2_D: 79.6 ± 15.7 pmol/L
	Nakamura (2001) [141]	Nursing home employees	77	Niigata, Japan (37° 48′ to 59′N)	Cross-sectional	42.1 ± 15.1	<50 nmol/L: 26%	Mean 1α, 25-(OH)_2_D: 111.1 ± 33.6 pmol/L
	Platz (2004) [142]	Health professionals	460	United States (37°09′N)	Case-control	59.7 ± 20.5		Mean 1α, 25-(OH)_2_D: 83.8 ± 17.0 pmol/L.
	Arya (2004) [143]	Hospital staff	92	Lucknow, 26.55°N, 80.59°E	Descriptive	30.7 ± 27.2	<50 nmol/L: 78.3%	Mean 1α, 25-(OH)_2_D: 97.4 ± 48.2 pmol/L
	Hanwell (2010) [144]	1. Hospital workers (in winter)	47	South Italy (latitude 40°N)	Descriptive	38.8 ± 29.0	<75 nmol/L: 87% <30 nmol/L: 53%	
		2. Hospital workers (in summer)	23			58.6 ± 16.5	<75 nmol/L: 78% <30 nmol/L: 4%	
	Beloyartseva [29]	Healthcare professionals	2119	India, various cities (20°59′N)	Descriptive	35.8 ± 26.5	<75 nmol/L: 15% <50 nmol/L: 79%	
	Plotnikoff (2012) [145]	Health care system employees	10,646	Minnesota, United States (46°73′ N)	Prospective observational	70.1 ± 34.0	<75 nmol/L: 31.9% <50 nmol/L: 28.9%	
	Porojnicu[146]	Hospital employees	105	Bucharest, Romania (45° N)	Descriptive		<80 nmol/L: 17% <50 nmol/L: 80%	
	Gannage-Yared (2014) [147]	Hospital employees	392	Beirut, Lebanon (33°89′N)	Descriptive	39.0 ± 19.7	< 75 nmol/L: 23.5% < 50 nmol/L: 71.4%	
	Skarphedinsdottir (2014) [148]	1. Anaesthesia health care staff	106	Reykjavik, Iceland (64°08′N) Madison, WI, USA (43°07′N)	Descriptive	70.5 ± 30.9	<75 nmol/L: 56.6% <50 nmol/L: 39.6%	
		2. Anaesthesia health care staff	124			70.0 ± 30.0	<75 nmol/L: 61.3% <50 nmol/L: 29%	

Occupations were categorized as indoor workers, outdoor workers, shiftworkers, coalminers, lead/smelter workers, medical residents, healthcare students, practising physicians, nurses and other healthcare professionals. Data on 25-(OH)D level and vitamin D deficiency as well as location (latitude) of study were extracted. Where necessary, additional notes and explanations are included

*Abbreviations:* 25-(OH)D = 25-hydroxyvitamin D; 1α, 25-(OH)_2_D = 1,25-dihydroxyvitamin D

Thirty-five of 71 articles described studies performed in health care workers (physicians, nurses, hospital employees, health sciences or medical students, and other health professionals). Three studies were performed in coal miners, 5 in lead/smelter workers and 6 in shiftworkers. Eleven of the 71 papers described groups of outdoor workers; 19 studies were performed in indoor or office-based workers. Some of the primary studies included subjects of more than one occupational setting and such studies were categorized under more than one occupational group depending on the occupations described by the authors, as shown in Table 1. Each occupational category that we examined had 3 or more primary studies, therefore permitting further quantitative analysis. Studies in which we could not establish a well-defined occupational setting were excluded (Table 1).

We extracted data from the included studies based on season of the year in which the study was conducted, assay type (measure of validity) and intra- and inter-assay CV (indication of reliability) in order to assess study quality; ‘unknown’ indicates that a study did not describe the parameter in question (Table 2). As shown in Table 2, 43% of included studies were of high quality, 37% were of medium quality, and 20% were of low quality. Regarding assay types, 40% of reports employed a radioimmunoassay (RIA) technique to assess serum vitamin D levels, 14% used a competitive protein binding assay, a chemiluminescence assay technique was employed in 13% of studies, 11% assayed vitamin D levels via the high performance liquid chromatography (HPLC) assay, while 4%, 2% and 1%, respectively, employed the enzyme-linked immunosorbent assay (ELISA), liquid chromatography and radioceptor techniques.

**Table 2:** Assay type, measure of coefficient of variation (reliability) and seasons of included studies

Category	Study ID	Assay type	Reliability	Season of year	Study quality
Outdoor Workers	Haddad and Chyu [102]	Competitive protein binding assay	CV: 8–14%	Spring, Summer	High
	Devgun [85]	Competitive protein binding assay	Inter-assay CV: 10%	All year	High
	Devgun [25]	Competitive protein binding assay	Inter-assay CV: 10.7%	All year	High
	Devgun [103]	Competitive protein binding assay	Inter-assay CV: 10.6%	Autumn, Winter	High
	Azizi [104]	Competitive protein binding assay	Unknown	All year	Medium
	Norsang [105]	RIA	Unknown	Autumn	Medium
	Azizi [91]	RIA	Unknown	Winter	Medium
	Choi [106]	RIA	Inter-assay CV: 11.7–12.5%	All year	High
	Roomi [55]	EIA	Inter-assay CV: 4.9%	All year	High
	Oh [107]	RIA	Unknown	Unknown	Low
	Bacchelv [27]	Unknown	Unknown	Unknown	Low
Indoor Workers	Devgun [85]	Competitive protein binding assay	Inter-assay CV: 10%	All year	High
	Devgun [25]	Competitive protein binding assay	Inter-assay CV: 10.7%	All year	High
	Maeda [108]	Immunoradiometric assay	Inter-assay CV: 16% (for lowest values; 3% for highest values) Intra-assay: 4.8%	Summer, Spring, Winter	High
	Gulvady [109]	RIA	Unknown	Unknown	Low
	Islam [110]	EIA	Inter-assay CV: 7% Intra-assay CV: 5.4%	Unknown	Medium
	Azizi [104]	Competitive protein binding assay	Unknown	All year	Medium
	Itoh [28]	RIA/EIA	Inter –assay CV: 21.9	Winter, Autumn	High
	Vu [111]	Chemiluminescent assay	Inter-assay CV: 6–9% Intra-assay CV: 3–6%	Summer, Winter	High
	Choi [106]	RIA	Inter-assay CV: 11.7–12.5%	All year	High
	Xiang [56]	HPLC-MS/MS tandem	Inter-assay CV: 6.9–9.5% Intra-assay CV: 2.77–3.2%	All year	High
	Cinar [112]	HPLC	Inter-assay CV: 3.4% Intra-assay: 4.3%	Summer, Winter	High
	Jeong [23]	Unknown	Unknown	Unknown	Low
	Sharma [113]	Chemiluminescent assay	Unknown	Unknown	Low
	Yosephin [114]	EIA	Unknown	Unknown	Low
	Roomi [55]	EIA	Inter-assay CV: 4.9%	All year	High
	Kwon [54]	EIA	Unknown	Winter, Spring	Medium
	Oh [107]	RIA	Unknown	Unknown	Low
	Bacchel [27]	Unknown	Unknown	Unknown	Low
Shiftworkers	Ward [41]	ELISA	Intra-assay CV: 5.5–7.2% (concentrations standardized according to mean of values from vitamin D External Quality Assurance Survey)	Unknown	Medium
	Itoh [28]	RIA	Intra- and inter-day variation: 4.3–7%	Summer	High
	Kim [115]	RIA	Unknown	All year	Medium
	Jeong [23]	Unknown	Unknown	Unknown	Low
	Kwon [54]	EIA	Unknown	Winter, Spring	Medium
	Romano [45]	Chemiluminescent assay	Unknown	Spring	Medium
Lead/Smelter	Greenberg [79]	Competitive protein binding assay	Unknown	Unknown	Low
	Mason [78]	Radioreceptor assay	Inter-assay CV: 11.5% Intra-assay CV: 6.9%	Unknown	Medium
	Chalkley [39]	RIA	Unknown	Unknown	Low
	Kristal-Boneh [77]	Competitive protein binding assay	Inter-assay CV: 15.2% Intra-assay CV: 3.9%	Summer	High
	Potula [116]	RIA	Unknown	Unknown	Low
Coalminers	Shuster [82]	Competitive protein binding assay	Unknown	Spring, Summer	Medium
	Shuster [81]	Competitive protein binding assay	Unknown	Winter, Autumn	Medium
	Sarikaya [83]	ELISA	Unknown	Unknown	Low
Healthcare Students	Maeda [108]	Immunoradiometric assay	Inter-assay CV: 16% (for lowest values; 3% for highest values) Intra-assay CV: 4.8%	Summer, Spring, Winter	High
	Gonzalez-Padilla [117]	Immunochemiluminescence	Inter-assay CV: 7.1–10% Intra-assay CV: 3–4.5%	Unknown	Medium
	Kaehler [118]	Electro-chemoluminescence	Unknown	Spring	Medium
	Al-Elq [119]	Chemiluminescent assay	Unknown	Winter	Medium
	Manickam [120]	Chemiluminescent assay	Inter-assay CV: 13.9% Intra-assay CV: 10.8%	Unknown	Medium
	Zabihiyeganeh [121]	RIA	Inter-assay CV: 6.4% Intra-assay CV: 5.6%	Autumn	High
	Milovanovic [122]	Unknown	Unknown	Spring, Summer	Low
Medical Residents	Haney [61]	RIA	Unknown	Autumn, Spring	Medium
	Maeda [108]	Immunoradiometric assay	Inter-assay CV: 16% (for lowest values; 3% for highest values) Intra-assay: 4.8%	Summer, Spring, Winter	High
	Premaor [123]	Chemiluminescent assay	Intra-assay CV: 6%	Autumn	High
	Multani [76]	RIA	Inter-assay CV: 6.49% Intra-assay CV: 3.85%	Spring, Summer	High
	Singh [124]	RIA	Unknown	Winter, Summer	Medium
	Growdon [125]	RIA	Inter-assay CV: 6.2–12.5% Intra-assay CV: 4.4–8.3%	Winter	High
	Mendoza [126]	Chemiluminescent assay	Inter-assay CV: 2.1% Intra-assay CV: 4.1%	Summer	High
	Ramirez-Vick [127]	LC-MS/MS	Unknown	Spring, Winter	Medium
Practising Physicians	Gann [128]	RIA	Intra-assay CV: 8.1%	Unknown	Medium
	Goswami [129]	RIA	Inter-assay CV: 13% Intra-assay CV: 8%	Winter, Summer	High
	Kramm [130]	HPLC	Unknown	Unknown	Low
	Mahdy [75]	Unknown	Unknown	Unknown	Low
	Lee [131]	RIA	Intra-assay CV: 13.8%	Summer, Autumn	high
	Haliloglu [132]	HPLC	Inter-assay CV: 3.1–4.7% Intra-assay CV: 0.7–4.9%	Winter, Summer	High
	Munter [133]	Chemiluminescent assay	Unknown	Unknown	Low
Nurses	Platz [134]	RIA	Intra-assay CV: 7.5%	Unknown	Medium
	Eliassen [135]	RIA	Overall CV: 10.7% and 6%	Unknown	Medium
	Hattapornsawan [136]	LC-MS/MS	Inter-assay CV: 6.3% Intra-assay CV: 5%	Unknown	Medium
	Wallingford [137]	RIA	Intra-assay CV: 0.99%	All year	High
	Wang [138]	RIA	Overall CV: 10.7% and 6%	Unknown	Medium
	Haliloglu [132]	HPLC	Inter-assay CV: 3.1–4.7% Intra-assay CV: 0.7–4.9%	Winter, Summer	High
	Bertrand [139]	High affinity protein-binding assay or RIA	Overall CV: 17.6% and 6%	Unknown	Medium
	Madani [140]	ELISA	Unknown	Summer	Medium
Other Healthcare Professionals	Platz [59]	RIA	Intra-assay CV: 6.7%	Unknown	Medium
	Nakamura [141]	HPLC	Inter-assay CV: 2.6–4.2%	Winter	High
	Platz [142]	RIA	Intra-assay CV: 5.4–5.6%	All year	High
	Arya [143]	RIA	Inter-assay CV: 8.4%	Unknown	Medium
	Hanwell [144]	RIA	Inter-assay CV: 12% Intra-assay CV: 7.2%	Winter, Summer	High
	Beloyartseva [29]	RIA	Unknown	Winter	Medium
	Plotnikoff [145]	Chemiluminescent assay	CV: 9.8–12.5%	Winter, Spring	High
	Porojnicu [146]	HPLC	Inter-assay CV: 12%	Winter	High
	Gannage-Yared [147]	Chemiluminescent assay	Inter- and Intra CV: <12%	All year	High
	Skarphedinsdottir [148]	HPLC	Unknown	Spring	Medium
	Haliloglu [132]	HPLC	Inter-assay CV: 3.1–4.7% Intra-assay CV: 0.7–4.9%	Winter, Summer	High

The assay type, coefficient of variation and the season of each study were extracted to assess the methodological quality of each study

*Abbreviations*: *CV* coefficient of variation, *RIA* radioimmunoassay, *LC-MS/MS* liquid chromatography-mass spectrometry/mass spectrometry, *ELISA* enzyme-linked immunosorbent assay, *EIA* electrochemiluminescence immunoassay, *HPLC* high performance liquid chromatography

### Indoor/office and outdoor workers

**Fig. 1:**
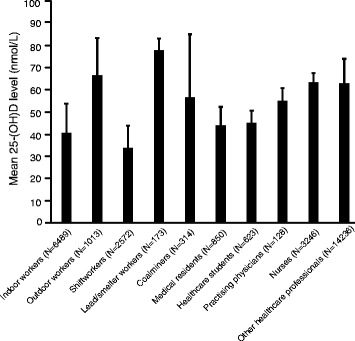
25-hydroxyvitamin D (25-(OH)D) levels in different occupational groups. Data represent the weighted means pooled from the means of the included studies obtained for each occupational category. Error bars represent pooled standard error of means computed as $SEp= Sp\sqrt{\frac{1}{n_1}+\frac{1}{n_2}}$, where Sp is pooled variance, n_1_ represents sample size of group 1, and n_2_ represents sample size of group 2

**Fig. 2:**
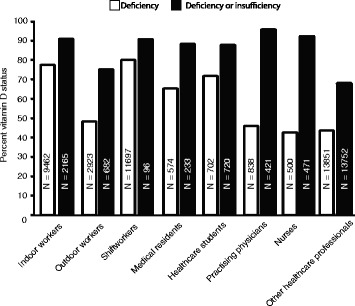
Percent vitamin D status in different occupational groups. Vitamin D deficiency (white bars) was defined according to the Endocrine Society’s (ES) categorization as a serum level of 25-(OH)D ≤ 50 nmol/L (20 ng/ml). Each white bar graph represents % of subjects of each group with a serum 25-(OH)D ≤ 50 nmol/L. The black bars represent percent vitamin D deficiency or insufficiency in different occupational groups. Vitamin D insufficiency was defined based on the ES’s criteria, which indicates a serum level of 25-(OH)D ≤ 75 nmol/L (30 ng/ml) as insufficient. Each black bar graph represents the % of subjects of each group with a serum 25-(OH)D level ≤ 75 nmol/L. The numbers within the bars, N, represent the total number of subjects contributing to each category for whom vitamin D deficiency, insufficiency, or sufficiency could be determined

We compared vitamin D level and the proportion of workers with deficiency/insufficiency between indoor and outdoor workers. As shown in Fig. 1 (and Additional file 4: Figure S1), the mean vitamin D level was significantly lower in indoor/office workers compared to outdoor workers (40.6 ± 13.2 nmol/L vs. 66.6 ± 16.7 nmol/L; *p* < 0.0001). Figure 2 demonstrates that 78% of indoor workers were vitamin D deficient in contrast to only 48% of outdoor workers who were vitamin D deficient. There was also a statistically significant difference between indoor and outdoor workers in the proportion who were vitamin D deficient or insufficient: 91% of indoor workers had vitamin D levels below 75 nmol/L versus 75% of outdoor workers (*p* < 0.01) (Fig. 2). Indoor workers had significantly elevated RRs of 1.23 (95% CI: 1.22 to 1.24) and 1.24 (95% CI: 1.22 to 1.25), to develop vitamin D deficiency and insufficiency, respectively (Tables 3 and 4). Outdoor workers had a significantly reduced susceptibility to vitamin D deficiency (RR: 0.77; 95% CI: 0.75 to 0.79) but no significantly different risk to develop vitamin D insufficiency (RR: 1.02; 95% CI: 0.98 to 1.07) (Tables 3 and 4).

**Table 3:** Occupational groups, % deficiency, and relative risk

Occupational group	Number of subjects	Number of vitamin D deficient subjects	% deficiency (25-(OH)D < 50 nmol/L)	Relative risk
All groups (total)	46,426	29,255	63.0	1.00 (baseline)
Indoor workers	12,204	9462	77.5	1.23 (95% CI: 1.22 to 1.24)
Outdoor workers	6060	2923	48.2	0.77 (95% CI: 0.75 to 0.79)
Shiftworkers	11,697	9354	80	1.27 (95% CI: 1.26 to 1.28)
Medical residents	574	375	65.3	1.04 (95% CI: 0.97 to 1.10)
Healthcare students	702	504	71.6	1.14 (95% CI: 1.09 to 1.19)
Practising physicians	838	386	46.1	0.73 (95% CI: 0.68 to 0.78)
Nurses	500	213	42.3	068 (95% CI: 0.61 to 0.75)
Other healthcare workers	13,851	6038	43.6	0.69 (95% CI: 0.68 to 0.71)

Vitamin D deficiency was defined as 25-(OH)D < 50 nmol/L

*Abbreviation: CI* confidence interval

**Table 4:** Occupational groups, combined % insufficiency and deficiency, and relative risk

Occupational group	Number of subjects	Number of vitamin D insufficient subjects	% insufficiency (25-(OH)D < 75 nmol/L)	Relative risk
All groups (total)	18,704	13,735	73.4	1.00 (baseline)
Indoor workers	2383	2165	90.9	1.24 (95% CI: 1.22 to 1.25)
Outdoor workers	682	513	75.2	1.02 (95% CI: 0.98 to 1.07)
Shiftworkers	96	91	90.8	1.24 (95% CI: 1.16 to 1.32)
Medical residents	233	205	88.2	1.20 (95% CI: 1.15 to 1.26)
Healthcare students	720	632	87.8	1.20 (95% CI: 1.16 to 1.23)
Practising physicians	421	403	95.7	1.30 (95% CI: 1.28 to 1.33)
Nurses	417	385	92.3	1.26 (95% CI: 1.22 to 1.29)
Other healthcare workers	13,752	9341	67.9	0.93 (95% CI: 0.91 to 0.94)

Vitamin D insufficiency was defined as 25-(OH)D < 75 nmol/L

*Abbreviation: CI* confidence interval

To determine the effect of latitude on vitamin D status and deficiency, the latitudes of the various study locations were obtained and plotted against mean vitamin D levels, % deficiency and % non-vitamin D sufficient (e.g. deficient or insufficient). On average, at any given latitude, the mean vitamin D levels of outdoor workers were higher than values seen in indoor workers (Additional file 3: Figure S4B). In general, a higher proportion of indoor workers were vitamin D deficient compared to outdoor workers (Additional file 5: Figure S5B). That vitamin D deficiency or insufficiency was higher in indoor workers relative to outdoor workers was not dependent on study location (Additional file 6: Figure S6B).

### Shiftworkers

Our analysis demonstrated that the impact of shiftwork on vitamin D status was considerable. Of all the occupational categories that were studied, shiftworkers had the lowest average levels of serum vitamin D (33.8 ± 10.1 nmol/L) (Fig. 1). About 80% of shiftworkers had serum vitamin D levels ≤50 nmol/L, indicating vitamin D deficiency (Fig. 2). Of the 6 studies on shiftworkers, only 1 study [45] reported % vitamin D insufficiency in addition to deficiency. In this one study, which was conducted at latitude 45`30° N, about 91% of subjects were found to be vitamin D deficient or insufficient [45] (Fig. 2). As shown in Tables 3 and 4, shiftworkers had the highest risk to develop vitamin D deficiency (RR: 1.27; 95% CI: 1.26 to 1.28) and a RR of 1.24 (95% CI: 1.16 to 1.32) to develop vitamin D insufficiency.

### Lead and smelter workers

Five studies of 183 subjects reported on lead and smelter workers. The mean vitamin D level of lead/smelter workers was 77.8 ± 5.4 nmol/L; they represented the occupational group with the highest vitamin D level among all the occupational categories we investigated (Fig. 1). None of the studies presented data on the proportion of subjects who were either vitamin D deficient or insufficient. All the included studies on lead/smelter workers also measured circulating levels of 1α, 25-(OH)_2_D, the active metabolite of vitamin D. In a sub-analysis, we showed that the average level of 1α, 25-(OH)_2_D in lead/smelter workers was 139.73 ± 57.51 (mean ± SD) pmol/L (Table 1).

### Coalminers

There was a paucity of studies that investigated the status of vitamin D in coalminers in the literature. Only 3 studies assessed vitamin D levels in coalminers, with an overall total of 314 subjects. The average serum vitamin D level in coalminers was 56.6 ± 28.4 nmol/L (Table 1). In order to evaluate the impact of the type of mining on vitamin D status, we further divided coalminers into underground and surface miners. Our analysis revealed that there was no statistically significant difference in average vitamin D levels between underground and surface miners (57.8 ± 11.7 vs. 52.4 ± 12.4 nmol/L, *p* = 0.78) (Additional file 7: Figure S2). None of the three studies analyzed reported data on the number of subjects that were vitamin D deficient or insufficient.

### Healthcare workers

We found that the overall mean serum 25-(OH)D level of all healthcare workers was 61.6 ± 11.0 nmol/L (data from 19,083 study subjects from 35 different studies). Among healthcare workers, our analysis demonstrated that medical residents and healthcare students have the lowest level of circulating vitamin D (44.0 ± 8.3 nmol/L and 45.2 ± 5.5 nmol/L, respectively) and there was no statistically significant difference (*p* = 0.9) between these two sub-groups (Additional file 8: Figure S3). Additionally, 65% and 72% of medical residents and healthcare students, respectively, were vitamin D deficient (Fig. 2). According to Tables 3 and 4, medical residents had RR of 1.04 (95% CI: 0.97 to 1.10) of vitamin D deficiency and the RR was 1.14 for healthcare students (95% CI: 1.09 to 1.19). With respect to vitamin D insufficiency, medical residents had a RR of 1.20 (95% CI: 1.15 to 1.26) and the RR was 1.20 (95% CI: 1.16 to 1.23) for healthcare students.

Seven studies reported on vitamin D in practising physicians, but only three studies comprising 128 subjects reported 25-(OH)D levels; the mean 25-(OH)D level was 55.0 ± 5.8 nmol/L (Table 1 and Additional file 8: Figure S3). Relative to medical residents and healthcare students, the higher level of 25-(OH)D in practising physicians was statistically significant (*p* < 0.001 for the comparison against each group). Vitamin D deficiency in practising physicians, reported in four studies (835 subjects) was 46%, significantly lower than that seen in medical residents and healthcare students (*p* < 0.001 and *p* < 0.001, respectively) (Fig. 2). As shown in Tables 3 and 4, practising physicians had a RR of 0.73 (95% CI: 0.68 to 0.78) and 1.30 (95% CI: 1.28 to 1.33) of vitamin D deficiency and insufficiency, respectively.

The average serum 25-(OH)D level in 3246 nurses from 8 studies was 63.4 ± 4.2 nmol/L (Fig. 1 and Additional file 8: Figure S3). The difference between mean vitamin D status in nurses compared to medical residents, healthcare students or practising physicians, was statistically significant (*p* < 0.0001 vs. both medical students and healthcare students; *p* < 0.01 vs. practising physicians; Fig. 1). Furthermore, our analysis showed that 43% of 500 nurses were deficient in serum 25-(OH)D (Fig 2). The proportion of nurses deficient in vitamin D was not significantly different compared to practising physicians (*p* = 0.6), but differed significantly when compared with medical residents and healthcare students (*p* < 0.001 and *p* = 0.02, respectively; Fig. 2). Nurses had a RR of 0.68 (95% CI: 0.61 to 0.75) to develop vitamin D deficiency and a RR of 1.26 (95% CI: 1.22 to 1.29) of insufficiency (Tables 3 and 4).

The final sub-division of the healthcare category was the group of employees we termed ‘other healthcare employees’, which comprised all employees in healthcare who were not specifically identified as nurses, physicians, medical residents or healthcare students. Eleven studies of 14,236 subjects reported on vitamin D level in this group of workers and the average vitamin D level was 63.0 ± 11.0 nmol/L, similar to values obtained in nurses, as described above. Similar to nurses, 43% of the other healthcare employees group were vitamin D deficient (Fig 2). Other healthcare workers had a RR of 0.69 (95% CI: 0.68 to 0.71) of vitamin D deficiency and a RR of 0.93 (95% CI: 0.91 to 0.94) for vitamin D insufficiency (Tables 3 and 4). The vitamin D level and proportion with deficiency in the ‘other healthcare employees’ differed significantly when compared with either medical residents (*p* < 0.0001), healthcare students (*p* < 0.0001) or practising physicians (*p* < 0.001), but not nurses (*p* = 0.9). Geographical latitude did not affect vitamin D levels and prevalence of deficiency or insufficiency among healthcare professionals (Additional file 3: Figure S4C; Additional file 5: Figure S5C; Additional file 6: Figure S6C).

**Fig. 3:**
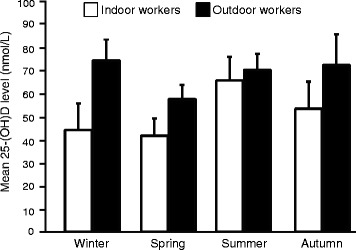
Effect of seasons on 25-(OH)D level in indoor (white bars) and outdoor (black bars) workers. Data represent mean ± standard error of the mean of each season for the given occupational group

Most of the studies did not provide data on the season of the year in which the studies were conducted; thus, we could not perform a quantitative analysis on the seasonal effect on vitamin D levels across the various occupational categories. However, enough studies on indoor and outdoor workers reported on the seasonal effect on vitamin D levels to enable us to conduct quantitative comparisons between these two groups. As shown in Fig. 3, regardless of the season, the mean level of 25-(OH)D in outdoor workers was different compared to indoor workers. Among outdoor workers the level of vitamin D was lowest in the spring (57.7 ± 6.2 nmol/L) compared to the winter (74.6 ± 9.0 nmol/L), the summer (70.5 ± 6.9 nmol/L) and the autumn (72.6 ± 13.3 nmol/L). Additionally, the 25-(OH)D levels in summer and autumn in outdoor workers were significantly different from levels found in winter (*p* < 0.0001) and spring (*p* < 0.0001). Surprisingly, values in the winter were comparable to the summer and autumn values in outdoor workers. In indoor workers, the highest value of vitamin D was observed in the summer (65.8 ± 10.3 nmol/L) and the lowest in the spring (41.8 ± 7.4 nmol/L) and winter (44.3 ± 11.6 nmol/L). When compared to autumn values (53.5 ± 11.8 nmol/L), the level of summertime vitamin D among indoor workers were significantly different (*p* < 0.0001).

### General observations on the studies

In general, serum 25-(OH)D levels in all occupational groups examined were below the optimal level as recommended by the ES, with the exception of lead/smelter workers. Lead/smelter workers had the highest level of serum vitamin D (77.8 ± 5.4 nmol/L) (Fig. 1) and were the only group whose average level was in the sufficient range. The average levels of vitamin D in outdoor workers, coalminers, practising physicians, nurses and other healthcare employees were in the insufficient range (25-(OH)D of 50–75 nmol/L) (Fig. 1). In indoor workers, shiftworkers, medical residents and healthcare students the average 25-(OH)D levels were in the deficient range (≤ 50 nmol/L). Average vitamin D levels were lowest (33.8 ± 10.1 nmol/L) among shiftworkers (Fig. 1 and Table 1).

When the various occupational groups were analysed with regard to the percentage with vitamin D deficiency, 80% of shiftworkers were vitamin D deficient and this group demonstrated the largest proportion of subjects in the deficient range (Fig. 2). They were followed closely by indoor workers and healthcare students with 77% and 72% vitamin D deficiency, respectively. Overall, outdoor workers, practising physicians, nurses and other healthcare employees all had proportions with vitamin D deficiency below 50% (Fig. 2).

The two occupational groups with the lowest proportion of combined deficiency or insufficiency were outdoor workers and other healthcare employees (75% and 68%, respectively) (Fig. 2). For practising physicians, about 96% were vitamin D deficient or insufficient. Likewise, indoor workers, shiftworkers and nurses all had about 90% deficiency or insufficiency.

The average serum vitamin D levels and prevalence of vitamin D deficiency or insufficiency (Additional file 3: Figure S4A; Additional file 5: Figure S5A; Additional file 6: Figure S6A) were not dependent on geographical location.

## Discussion

The global prevalence of vitamin D deficiency has reached an alarming proportion. This trend has elicited a significant amount of research interest to elucidate the potential causes of vitamin D deficiency and insufficiency in order to advance interventional strategies to ameliorate the associated risks [2, 46]. Several studies have demonstrated that populations worldwide, including those located in sunny regions of the world, are at risk of vitamin D deficiency [47, 48]. Some vulnerable demographic groups of the population, including pregnant women [49, 50], the elderly [51], hospitalized patients and other institutionalized groups [12, 52, 53], and certain occupations [45, 54–56] who are predisposed to receive low or no exposure to sunshine, may be at a greater risk of developing vitamin D inadequacy.

In general, we found that vitamin D levels in most occupational groups fell well below the levels considered optimal for health [13, 57]. Additionally, we observed a higher prevalence of vitamin D deficiency in all occupational groups examined than the reported population burden of vitamin D deficiency in multiple populations, suggesting that workers may be particularly vulnerable to vitamin D deficiency [23, 41]. For instance, 25-(OH)D among US subpopulations demonstrated that adult women had mean serum 25-(OH)D levels of 62 nmol/L and 75 nmol/L, respectively, in winter and summer [58]. Adult men had mean serum 25-(OH)D of 70 nmol/L and 82 nmol/L in winter and summer, respectively [58]. In contrast, Platz et al. [59] reported that among healthcare professionals in the US, the mean serum 25-(OH)D was 46 nmol/L. The prevalence of vitamin D deficiency was 30% [60] in the adult population in the US compared to 38% among medical residents [61, 62]. Other determinants of vitamin D deficiency include culture [63], geography [64, 65], genetics [66], disease states [67], diet [6] and age [68], and such other determinants will have to be considered alongside occupation as part of a comprehensive assessment of vitamin D status.

Among the occupations considered in the present study, indoor workers and shiftworkers demonstrated very low levels of serum 25-(OH)D and high rates of vitamin D deficiency and insufficiency. The relative risks of vitamin D deficiency and insufficiency in these two worker groups were also very high compared to the other worker groups. Indoor workers spend a high proportion of their working hours indoors without sunshine exposure. Additionally, indoor workers working conventional working hours would be expected to get their sunlight exposure during mornings and evenings, when sunlight intensity is relatively low. Since vitamin D is produced through sunshine and adequate UV exposure, sunlight deprivation in indoor workers may put them at greater risk of developing vitamin D deficiency and its accompanying health risks. Consistent with this assumption, our study showed that workers in an indoor setting displayed a lower level of vitamin D relative to their outdoor counterparts. Further, medical residents and healthcare students, who spend a considerable amount of time indoors, had vitamin D levels almost identical to the level of vitamin D observed in specified indoor workers (Fig. 1).

Our study also demonstrated that shiftworkers are at the highest risk of vitamin D deficiency or insufficiency when compared with other occupational groups. Shiftworkers make up about 20% of the workforce in developed countries [69]. Shift work may include rotational daytime shifts or overnight shifts. Kimlin and Tenkate [70] reported that workers with permanent night shifts receive less exposure to daytime solar UVB. This may result in a lower vitamin D level in shiftworkers relative to the general population. Additionally, shiftworkers with low sunlight exposure may depend on exogenous vitamin D for proportionately more of their total vitamin D requirements. These hypotheses are consistent with the findings of the present review, which revealed that shiftworkers had a low vitamin D level with a comparatively large proportion of workers with deficiency or insufficiency. The low level of serum 25-(OH)D seen in shiftworkers has been suggested to be associated with their predisposition to various diseases including cancers [71], musculoskeletal disorders, and cardiovascular disease [2, 72, 73].

The interpretation of our analysis is complicated by the fact that shiftworkers may work at various times of the day in a fixed or rotating pattern. Additionally, shiftworkers may have a greater proportion of their nonworking hours during daylight periods, and spend more time outdoors during nonworking days than indoor office workers. Another factor is that shiftworkers may be outdoor or indoor workers. Furthermore, shiftworkers may differ in dietary intake, use of vitamin D supplements, or other lifestyle factors from non-shiftworkers, and considerable variability may exist with regard to the amount of time spent outdoors. Nevertheless, a compelling finding from the present review is that shiftwork appears to be a strong predictor for vitamin D deficiency.

Another determining factor shown to impact vitamin D status in shiftworkers is the season during which vitamin D levels are measured. Ito et al. [28] demonstrated that, during the summer, the amount of ambient solar UVB can compensate for time confined working indoors in shiftworkers. However, in the winter, vitamin D levels were lower in shiftworkers who work fixed night shifts compared to the shiftworkers who work strictly daytime shifts. This finding was confirmed by Romano et al. [45], who showed that nighttime shiftworkers had lower vitamin D levels compared with daytime workers during spring. Taken together, these observations suggest that, although shiftworkers may be at greatest risk of vitamin D deficiency, spending sufficient time outside and obtaining sufficient UV exposure has the potential of alleviating this risk.

The lifestyle and nature of work of many healthcare professionals may suggest less opportunity to be exposed to daytime solar UVB. Thus, it is reasonable to assume that healthcare employees would be at risk of vitamin D deficiency. Additionally, working hours may be particularly long during the early training period for most healthcare professionals, where sun deprivation due to long working hours can be exacerbated by additional time spent indoors studying. Consistently, average vitamin D levels in healthcare students and medical residents were shown in the present review to be in the deficient range, and average serum vitamin D levels were significantly lower than those of practicing physicians, nurses, or other healthcare professionals. Indeed, the average serum vitamin D levels in medical residents and healthcare students were close to those of specified indoor workers. For healthcare professionals, our study revealed a high prevalence of vitamin D deficiency among healthcare students and medical residents. This is alarming, as students and residents are generally young adults, and vitamin D deficiency during early adulthood may decrease peak bone density and lead to an increased risk of osteopenia or osteoporosis in later life, as well as other long-term health impacts associated with suboptimal vitamin D status [74]. Additionally, vitamin D sufficiency in young healthcare professionals may be a surrogate marker for other healthy behaviours (e.g. outdoor exercise and good nutrition), and a high burden of vitamin D deficiency in trainees in the health disciplines should prompt enhanced educational measures on the importance of adequate vitamin D, as well as an examination of the underlying training-related factors which may contribute to vitamin D deficiency.

Practising physicians, nurses and other healthcare workers had average serum vitamin D levels significantly higher than students and residents, although the average vitamin D levels in all three groups were still in the insufficient range. The prevalence of vitamin D deficiency or insufficiency was also very high among all healthcare professionals with the exception of the other healthcare employee group. The latter group comprised employees with diverse work environments and lifestyles, which suggests that some may have more exposure to solar UVB not considered typical of many healthcare professionals. This could account for the relatively low prevalence of vitamin D deficiency or insufficiency in the other healthcare employees group.

The large proportion of studies on healthcare workers may in part reflect the use of healthcare workers as a convenience sample of young, presumed healthy individuals. Additionally, several large population-based studies in health professionals (e.g. Nurses’ Health Study, Physicians’ Health Study) have been performed, and publications arising out of these cohorts are included in our analysis. However, it is concerning that among healthcare workers, such a high prevalence of vitamin D deficiency exists. This may reflect a number of occupational factors including long working hours mainly indoors [61, 62], shiftwork and a tendency for healthcare workers to neglect their own health [29, 75, 76]. The extent to which healthcare workers, in general, are aware of their own vitamin D status is unknown, and it is conceivable that targeted interventions aimed at identifying and treating vitamin D deficiency in health care workers may be beneficial. Of note, some healthcare professions (e.g. nursing) have a high proportion of female workers of childbearing age, and there may be ancillary health benefits beyond those to healthcare workers themselves.

Surprisingly, our data also demonstrate that among healthcare professionals, only healthcare students had an increased risk to develop vitamin D deficiency. However, all healthcare professionals had an elevated risk of vitamin D insufficiency, in keeping with the 25-(OH)D levels in these worker categories.

Lead exposure, either short- or long-term, has the potential to influence the metabolism of vitamin D [77, 78]. The impact of lead on serum 1α, 25-(OH)_2_D levels is attributed to the inhibitory effect of lead on cytochrome P450 in the proximal tubules of the kidney, which mediates the hydroxylation of 25-(OH)D to the dihydroxy metabolite. In adults occupationally exposed to lead [78], Mason et al. demonstrated increased serum 1α, 25-(OH)_2_D levels in lead-exposed workers compared with a referent group who were not occupationally exposed to lead. In contrast, Greenberg et al. [79] did not demonstrate any effect of lead exposure on serum 1α, 25-(OH)_2_D levels.

Of all the occupational groups considered in our study, lead/smelter workers had the highest level of circulating 25-(OH)D. The mean 1α, 25-(OH)_2_D levels found in lead/smelter workers in our study was 139.73 ± 57.51 pmol/L, consistent with the average serum 1α, 25-(OH)_2_D levels found in the general population [80]. This may imply that the lead/smelter workers did not demonstrate compromised renal hydroxylase activity. The number of studies on lead/smelter workers and the number of study participants were, however, very few relative to the other occupational groups examined except coalminers. Moreover, the studies were older and the nutrition of the subjects at the time may have been different compared to subjects in more recent studies. Furthermore, the studies on lead/smelter workers presented in this report also are of low quality; they did not demonstrate any relationship between serum 25-(OH)D levels and blood lead levels. Thus, modern and high quality studies that account for all confounders of the relationship between lead exposure and serum 25-(OHD) and 1α, 25-(OH)_2_D levels are warranted to establish if there is an association between lead exposure and vitamin D status.

Likewise, the literature is lacking in high quality studies that describe the relationship between coalminers and serum vitamin D levels. Our review found only three reports [81–83] comprising 314 subjects. Coalminers can be underground miners or surface miners. Underground miners experience reduced exposure to sunlight and, following on from the previous discussion, may be at increased risk of low vitamin D status compared to surface miners. Surprisingly, our findings revealed that vitamin D status in underground miners was not significantly different from surface miners. The lack of appreciable difference in these two group of miners may mean that exposure of the underground group to sunlight in-between shifts could be sufficient to maintain serum vitamin D levels. In keeping with this view, Shuster et al. [81, 82] showed that, in the summer and winter seasons, serum vitamin D levels were not significantly different between underground and surface workers. However, in the summer months, serum vitamin D levels were higher than the corresponding levels seen during the winter months. These findings were in agreement with those demonstrated by Sarikaya et al. [83] in underground and surface miners.

The angle at which the sun rays impact the skin, which is a function of latitude, determines the amount of vitamin D production [64]. The more oblique the angle, the lesser the amount of vitamin D synthesized [84]. At latitudes beyond 35°, vitamin D production declines [65]. However, geography did not seem to be an obvious determinant of the difference in vitamin D level, as evidenced in the apparent lack of impact of latitude on vitamin D levels (Additional file 3: Figure S4A-C), and this is consistent with other published literature. A recent study in the US demonstrated that, for a large proportion of the year (March – October), serum vitamin D status was independent of geographical latitude [64]. This study further described that latitude becomes limiting only during the winter months (November – February). In our review, indoor vs. outdoor work and the amount of time exposed to sunlight seemed to be the dominant determinant of vitamin D levels.

Serum 25-(OH)D levels vary widely according to the season of the year in which the studies or collection of samples are conducted [25, 85], thus establishing season of the year as a confounder of serum vitamin D level [84]. Several lines of evidence suggest that there is a high variability in seasonal vitamin D levels across the globe [86, 87]. In a study on a normal Japanese population (adults without any abnormal biochemical data shown on routine medical check-up; in particular, those not suffering from parathyroid or calcium-related diseases, based on biochemical measurements and clinical assessment), Ono et al. [88] showed that mean serum 25-(OH)D levels were lowest in winter and spring, and peaked in the summer and the beginning of autumn. These findings were consistent with data from healthy postmenopausal women in New Zealand presented by Bolland et al. [89]. Accordingly, vitamin D deficiency was more prevalent in the spring and winter relative to the level of deficiency seen in summer and autumn [64]. These findings confirm the dependence of vitamin D status on season. Devgun et al. [85] also demonstrated in both indoor and outdoor workers that serum 25-(OH)D levels varied significantly according to season, being lowest in the spring and winter, and highest in the late summer and the beginning of autumn, in agreement with previous data [88, 89]. More importantly, they showed that vitamin D levels in outdoor workers were higher relative to indoor workers for all seasons but more pronounced in early winter.

In this systematic review, we could not perform a meta-analysis on all occupational groups to determine the effect of seasonality on vitamin D status because most of the included studies did not report on the season in which serum vitamin D level was assessed. We suggest that seasonality should be routinely reported in future studies on vitamin D levels to try to address this gap. Nonetheless, from the included studies which reported on seasons in indoor and outdoor workers, our data showed that vitamin D levels in outdoor workers were higher relative to indoor workers in all four seasons (Fig. 3). An unexpected finding in the present study was that the level of 25-(OH)D in the winter was comparable to the summer and autumn values in outdoor workers. Endogenous vitamin D synthesis is a function of UV radiation in the wavelength range of 280–320 nm, which in turn depends on season and latitude [64, 65, 85, 90]. At high latitudes (>35° N) UV radiation becomes almost negligible in the winter months, which consequently affects vitamin D production [85]. In contrast, at lower latitudes, UV radiation is not limiting during the winter months, which suggests that vitamin D synthesis can proceed all year long [64]. An alternative explanation may be that it is possible to accumulate sufficient vitamin D stores to get through the winter, but that the stores are depleted by springtime. Therefore, the lowest levels are seen in spring.

In the present study, the location of the studies that contributed the highest amount to the pooled mean 25-(OH)D level in outdoor workers in the winter were at latitudes <35° N [91]. Thus, outdoor workers in these regions may not experience huge seasonal variation in vitamin D synthesis compared to indoor workers. Together with the fact that there were few studies that contributed to the analysis of serum 25-(OH)D level dependence on season, this could account for the relatively high level of 25-(OH)D in outdoor workers during the winter season.

Most adults in the general population globally have vitamin D inadequacy [13, 46]. A significant proportion of the adult population in Europe, the US and Canada have vitamin D deficiency [2, 92]. Despite the relationship between sunlight exposure and vitamin D levels, vitamin D deficiency is reportedly also prevalent among populations living in sunny climates including the Middle East, Africa, Australia, India and South America [2, 93, 94]. Population-level prevalences of vitamin D deficiency have been reported as 59% in the Canadian population [95], 52% in the Danish population [96], and 40% in the US population [97, 98]. Our systematic review suggests that occupation is a major determinant that may contribute to suboptimal vitamin D levels and that workers in some occupations have lower average levels of vitamin D and a higher prevalence of deficiency compared to the general public. Indoor workers, shiftworkers, medical residents, healthcare students, practising physicians and coalminers have a particularly high prevalence of vitamin D deficiency. However, most occupational groups considered in this review, with the exception of lead/smelter workers, had a moderate to high burden of vitamin D deficiency or insufficiency.

### Clinical Implications and Recommendations

Although population-wide vitamin D deficiency is a global phenomenon, from the present systematic review it is clear that workers in some occupational categories are at a greater risk for vitamin D deficiency than others. Regular screening for vitamin D levels in shiftworkers and other specific groups of workers should be considered for future clinical practice guidelines and population health initiatives, while existing workplace wellness programs should incorporate education about the importance of adequate vitamin D levels, sunlight exposure and adequate nutritional intake of vitamin D-rich foods to prevent adverse health outcomes related to vitamin D deficiency. Additionally, for occupations predominantly based indoors, workers could, where appropriate, be encouraged to take intermittent breaks outside to expose the skin to UV light in order to promote cutaneous vitamin D synthesis [99, 100], and work schedules could be re-imagined to allow for such breaks, while of course avoiding excessive sunlight exposure.

### Limitations

To further the aim of a robust body of literature on the health effects of suboptimal vitamin D status, the academic community would benefit from a consensus as to what constitutes vitamin D deficiency. Due to an existing lack of agreement on the definition of vitamin D deficiency, combining data from studies where the study authors have used different definitions of adequate vitamin D status is challenging. In the present study, we used the ES’s definition of vitamin D deficiency and insufficiency. Other approaches, such as using the Institute of Medicine’s definitions, could also be justified. Agreement on what constitutes vitamin D insufficiency and deficiency will additionally enhance standardization of guidelines and interventional efforts targeted at at-risk occupational groups in the population.

Another limitation is the methodology employed in assaying serum vitamin D levels. In the present review, the majority of the included studies used the RIA technique to evaluate vitamin D status. Several lines of evidence have shown that there is a marked inter-laboratory variation in results obtained with this assay type, which could be as high as 30% [8, 13]. These variations need to be borne in mind when pooling data from different studies.

Serum vitamin D level is determined by latitude (geographical location), season (UVB), cultural traditions (clothing), diet and sex. The present review combines studies conducted at different latitudes, seasons and with subjects from diverse cultural backgrounds. This has the potential to overestimate or underestimate the influence of occupation on vitamin D. These differences may also create a high degree of heterogeneity between individual studies making conclusions derived from pooled data less reliable.

Our systematic review is also limited with regard to occupational detail, as we relied on what was reported by the primary study authors in their description of the subjects’ occupations or occupational categories.

### Recommendations for future studies

Based on the above limitations, we suggest that future studies measuring vitamin D status employ assay techniques with minimal inter-laboratory variations. One method, which has been shown to be consistently reproducible, is liquid chromatography [101]. In a review to compare different assays used to assess vitamin D status, it was demonstrated that liquid chromatography followed by tandem mass spectrometry produced the lowest variability across different laboratories [6]. It is recommended that the assay technique for the assessment of vitamin D levels should be standardized to enable ready comparison and meta-analysis.

Though medical students and residents may still be considered as a convenience sample, future studies employing this group as subjects should not presume that they are necessarily a population of “healthy” young adults.

Since season of the year is a major determinant of vitamin D levels, future studies should comment on the season in which the study is performed.

Future studies should also incorporate additional confounders such as measures of sunlight exposure and diet. Studies on shiftworkers should furthermore provide an indication of the type of shiftwork performed.

## Conclusions

Individuals who work predominantly indoors and shiftworkers are at risk of developing vitamin deficiency or insufficiency. Despite a lack of consensus on optimal levels of vitamin D for health, vitamin D insufficiency and deficiency are common in the occupational groups investigated, and some workers should be considered an at-risk group for vitamin D deficiency. Further high quality studies are needed to explore the relationship between occupation and vitamin D status. The assumption that trainees in the health care disciplines represent a convenience sample of “healthy” adults may not always be true. Guidelines on screening for vitamin D deficiency and supplementation strategies in vulnerable groups should include consideration of occupation.

## Additional files

Additional file 1: Search strategies. (DOCX 21 kb)

Additional file 2: Study selection. (DOC 57 kb)

Additional file 3: 25-(OH)D levels. **Figure S4A.** Effect of latitude on serum 25-(OH)D levels in all occupational groups examined. Each data point represents mean ± standard error of the mean of each included study. Inset: symbols and colors of each occupational subgroup. N/S: Northern/Southern hemisphere. **Figure S4B.** Comparison of serum 25-(OH)D levels in indoor and outdoor workers according to latitude. Each data point represents mean ± standard error of the mean of studies of outdoor and indoor workers included in the analysis. Inset: symbols and colors of each occupational subgroup. N/S: Northern/Southern hemisphere. **Figure S4C.** Comparison of serum 25-(OH)D levels in healthcare workers according to latitude. Each data point represents mean ± standard error of the mean of studies of each healthcare workers included in the analysis. Inset: symbols and colors of each occupational subgroup. N/S: Northern/Southern hemisphere. (ZIP 229 kb)

Additional file 4: 25-(OH)D levels in indoor and outdoor workers. **Figure S1.** 25-(OH)D levels in indoor and outdoor workers. Data represent pooled weighted mean ± pooled standard error of the mean for each group. * Statistically significant compared with indoor workers (*p < 0.0001*). (PDF 212 kb)

Additional file 5: Latitude and % vitamin D deficiency. **Figure S5A.** Effect of latitude on % vitamin D deficiency in all the occupational groups included in analysis. Percent vitamin D deficiency was defined as the number of subjects of a particular study with mean 25-(OH)D levels less than 50 nmol/L. Inset: symbols and colors of each occupational subgroup. N/S: Northern/Southern hemisphere. **Figure S5B.** Effect of latitude on % vitamin D deficiency in indoor and outdoor workers included in the analysis. Inset: symbols and colors of each occupational subgroup. N/S: Northern/Southern hemisphere. **Figure S5C.** Effect of latitude on % vitamin D deficiency in healthcare employees included in the analysis. Inset: symbols and colors of each occupational subgroup. N/S: Northern/Southern hemisphere. (ZIP 195 kb)

Additional file 6: Latitude and % vitamin D deficiency or insufficiency. **Figure S6A.** Effect of latitude on % vitamin D deficiency or insufficiency in all occupational groups examined. Percent vitamin D insufficiency was defined as the number of subjects of a particular study with mean 25-(OH)D levels less than 75 nmol/L. Inset: symbols and colors of each occupational group. N/S: Northern/Southern hemisphere. **Figure S6B.** Effect of latitude on % vitamin D deficiency or insufficiency in indoor and outdoor workers. Inset: symbols and colors of each occupational subgroup. N/S: Northern/Southern hemisphere. **Figure S6C.** Effect of latitude on % vitamin D deficiency or insufficiency in healthcare professionals. Inset: symbols and colors of each occupational subgroup. N/S: Northern/Southern hemisphere. (ZIP 189 kb)

Additional file 7: 25-(OH)D levels in underground and surface coalminers. **Figure S2.** 25-(OH)D levels in underground and surface coalminers. Data represent pooled weighted mean ± pooled standard error of the mean for each group. NS, no significant difference. (PDF 213 kb)

Additional file 8: 25-(OH)D levels in healthcare professionals. **Figure S3.** 25-(OH)D levels among different healthcare professionals. Data represent pooled weighted mean ± pooled standard error of the mean for each healthcare category. * Statistically significant compared to medical residents (*p < 0.05*). (PDF 222 kb)

## Abbreviations

1α, 25-(OH)_2_D: 1α, 25-dihydroxyvitamin D
25-(OH)D: 25-hydroxyvitamin D
CI: Confidence interval
CV: Coefficient of variation
EIA: Enzyme immunoassay
ELISA: Enzyme-linked immunosorbent assay
ES: Endocrine Society
Fig.: Figure
HPLC: High performance liquid chromatography
IQR: Interquartile range
l: Liter
ml: Milliliter
N: North
ng: Nanogram
nmol: Nanomole
pmol: Picomole
RIA: Radioimmunoassay
RR: Relative risk
SD: Standard deviation
SE: Standard error of the mean
UVB: Ultraviolet B
