# Supplementary material for: Vitamin D levels and deficiency with different occupations: a systematic review
Source: BMC Public Health. 2017 Jun 22;17:519. doi: 10.1186/s12889-017-4436-z (PMC5480134; doi:10.1186/s12889-017-4436-z)
Supplement: Supplementary file 1 — Search strategies. (DOCX 21 kb) [file 12889_2017_4436_MOESM1_ESM.docx]

**Additional File 1: Search Strategies**

**Databases searched:**

- EMBASE (OvidSP): 1974 to March 24, 2016
- Ovid MEDLINE(R) In-Process & Other Non-Indexed Citations, Ovid MEDLINE(R) Daily and Ovid MEDLINE(R): 1946 to March 24, 2016
- CINAHL Plus with Full Text (EBSCO interface): 1937 to March 24, 2016
- EBM Reviews – Ovid SP Cochrane Central Register of Controlled Trial: 1925 to March 24, 2016

**Full search strategy**

**Embase (OvidSP): 1974 to March 24, 2016**

Results: 1571

1. exp vitamin D/
2. vitamin D deficiency/
3. vitamin d.mp.
4. 1 or 2 or 3
5. exp named groups by occupation/
6. (job or jobs or worker* or occupation* or (shift* adj2 work*) or night shift*).mp.
7. exp employment/
8. occupation/ or career/ or job characteristics/ or vocation/
9. work/ or absenteeism/ or burnout/ or night work/ or return to work/ or work schedule/ or working time/
10. work environment/
11. "occupation and occupation related phenomena"/ or occupational science/
12. occupational health/ or employability/ or occupational exposure/ or occupational hazard/ or occupational safety/ or work capacity/
13. 5 or 6 or 7 or 8 or 9 or 10 or 11 or 12
14. 4 and 13
15. limit 14 to child
16. limit 14 to aged <65+ years>
17. 15 or 16
18. limit 17 to adult <18 to 64 years>
19. 14 not (17 not 18)

**Ovid MEDLINE(R) In-Process & Other Non-Indexed Citations, Ovid MEDLINE(R) Daily and Ovid MEDLINE(R): 1946 to March 24, 2016**

Results: 531

1. exp Vitamin D/

2. exp Vitamin D Deficiency/

3. vitamin d.mp.

4. 1 or 2 or 3

5. Occupations/

6. employment/ or workplace/

7. return to work/

8. work/ or work schedule tolerance/

9. "personnel staffing and scheduling"/ or workload/

10. Burnout, Professional/

11. Absenteeism/

12. Occupational Diseases/

13. exp occupational groups/

14. Occupational Health/

15. Occupational Exposure/

16. Occupational Medicine/

17. (job or jobs or worker* or occupation* or employee* or personnel or ((shift* or seasonal) adj2 work*) or night shift*).mp.

18. or/5-17

19. 4 and 18

20. limit 19 to ("all child (0 to 18 years)" or "all aged (65 and over)")

21. limit 20 to ("adult (19 to 44 years)" or "middle age (45 to 64 years)")

22. 19 not (20 not 21)

23. (((child* not childbearing) or infant* or newborn* or neonate* or teen* or adolescen* or pediatric* or paediatric* or elderly) not adult*).ti.

24. 22 not 23

**EBM Reviews - Cochrane Central Register of Controlled Trials (Ovid Interface): 1925 to March 24, 2016**

Results: 57

1. exp Vitamin D/

2. exp Vitamin D Deficiency/

3. vitamin d.mp.

4. 1 or 2 or 3

5. Occupations/

6. employment/ or workplace/

7. return to work/

8. work/ or work schedule tolerance/

9. "personnel staffing and scheduling"/ or workload/

10. Burnout, Professional/

11. Absenteeism/

12. Occupational Diseases/

13. exp occupational groups/

14. Occupational Health/

15. Occupational Exposure/

16. Occupational Medicine/

17. (job or jobs or worker* or occupation* or employee* or personnel or ((shift* or seasonal) adj2 work*) or night shift*).mp.

18. or/5-17

19. 4 and 18

20. (((child* not childbearing) or infant* or newborn* or neonate* or teen* or adolescen* or pediatric* or paediatric* or elderly) not adult*).ti.

21. 19 not 20

**CINAHL Plus with Full Text (EBSCOhost): 1937 to March 24, 2016**

Results: 346

S1: (MH "Vitamin D+") OR "vitamin d

S2: (MH "Named Groups by Occupation+") OR (MH "Occupations and Professions") OR (MH "Employment Status") OR (MH "Employment") OR (MH "Part Time Employment") OR (MH "Self Employment") OR (MH "Temporary Employment") OR (MH "Shiftwork") OR (MH "Personnel Staffing and Scheduling") OR (MH "Job Accommodation") OR (MH "Work Redesign") OR (MH "Work") OR (MH "Work Environment+") OR (MH "Burnout, Professional") OR (MH "Productivity") OR (MH "Absenteeism") OR (MH "Occupational Hazards") OR (MH "Occupational Exposure") OR (MH "Accidents, Occupational") OR (MH "Occupational Health") OR (MH "Occupational Medicine")

S3: job or jobs or worker* or occupation* or employee* or personnel or ((shift* or seasonal) n2 work*) or night shift*

S4: S1 AND (S2 OR S3)

S5: TI (((child* not childbearing) or infant* or newborn* or neonate* or teen* or adolescen* or pediatric* or paediatric* or elderly) not adult*)

S6: S4 NOT S5
