# Supplementary material for: Vitamin D levels and deficiency with different occupations: a systematic review
Source: BMC Public Health. 2017 Jun 22;17:519. doi: 10.1186/s12889-017-4436-z (PMC5480134; doi:10.1186/s12889-017-4436-z)
Supplement: Supplementary file 2 — Study selection. (DOC 57 kb) [file 12889_2017_4436_MOESM2_ESM.doc]

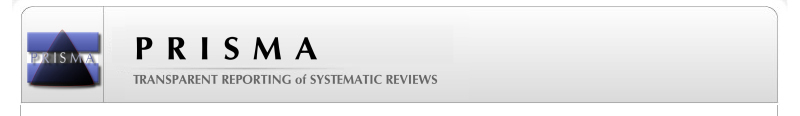
 **Flow Diagram – Study Selection**

**Screening**

**Included**

**Eligibility**

**Identification**

Records identified through database searching
(n=2505)

Additional records identified through other sources
(n=30)

Records after duplicates removed
(n=2008)

Records screened
(n=2008)

Records excluded
(n=1904)

Full-text articles assessed for eligibility
(n=104)

Full-text articles excluded (n=33):

No properly defined occupational group (n=12)

Articles in languages other than English or German (n=4)

Did not measure or report 25-(OH)D levels (n=9)

Unclear number of subjects (workers) (n=3)

Described mathematical models to predict 25-(OH)D levels, no levels measured (n=2)

Reported same workers as previous studies (n=2)

Pooled data from previous study (n=1)

Studies included in qualitative synthesis
(n=71)

Studies included in quantitative synthesis (meta-analysis)
(n = 66)
