# Supplementary figures and images for: Vitamin D levels and deficiency with different occupations: a systematic review
Source: BMC Public Health. 2017 Jun 22;17:519. doi: 10.1186/s12889-017-4436-z (PMC5480134; doi:10.1186/s12889-017-4436-z)

Supplementary Fig. 4A

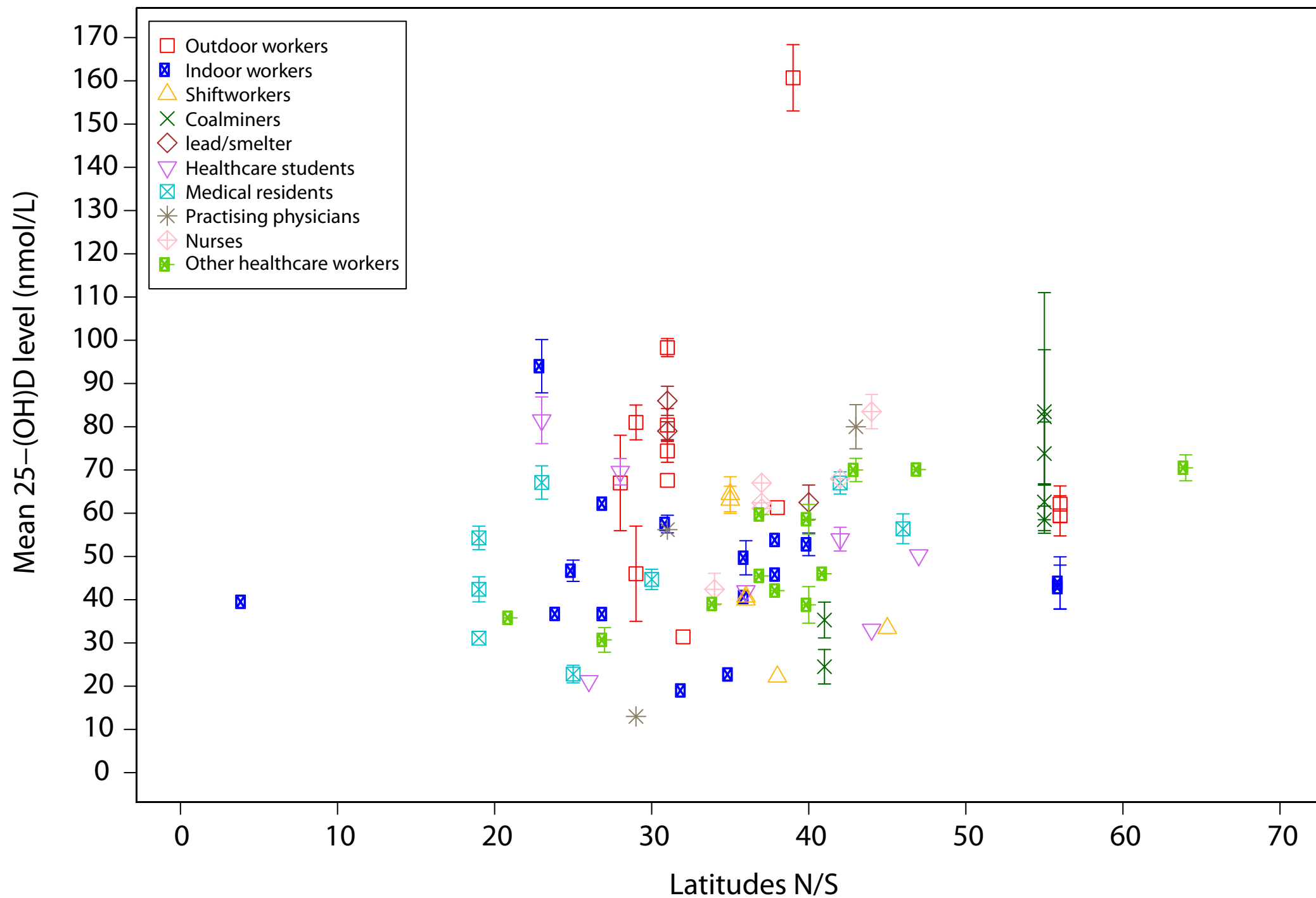

Supplement: Supplementary file 3 — 25-(OH)D levels. Figure S4A. Effect of latitude on serum 25-(OH)D levels in all occupational groups examined. Each data point represents mean ± standard error of the mean of each included study. Inset: symbols and colors of each occupational subgroup. N/S: Northern/Southern hemisphere. Figure S4B. Comparison of serum 25-(OH)D levels in indoor and outdoor workers according to latitude. Each data point represents mean ± standard error of the mean of studies of outdoor and indoor workers included in the analysis. Inset: symbols and colors of each occupational subgroup. N/S: Northern/Southern hemisphere. Figure S4C. Comparison of serum 25-(OH)D levels in healthcare workers according to latitude. Each data point represents mean ± standard error of the mean of studies of each healthcare workers included in the analysis. Inset: symbols and colors of each occupational subgroup. N/S: Northern/Southern hemisphere. (ZIP 229 kb) [file 12889_2017_4436_MOESM3_ESM.zip › Suppl Fig. 4A Latitude and 25-(OH)D levelsR3.pdf]

Supplementary Fig. 4B

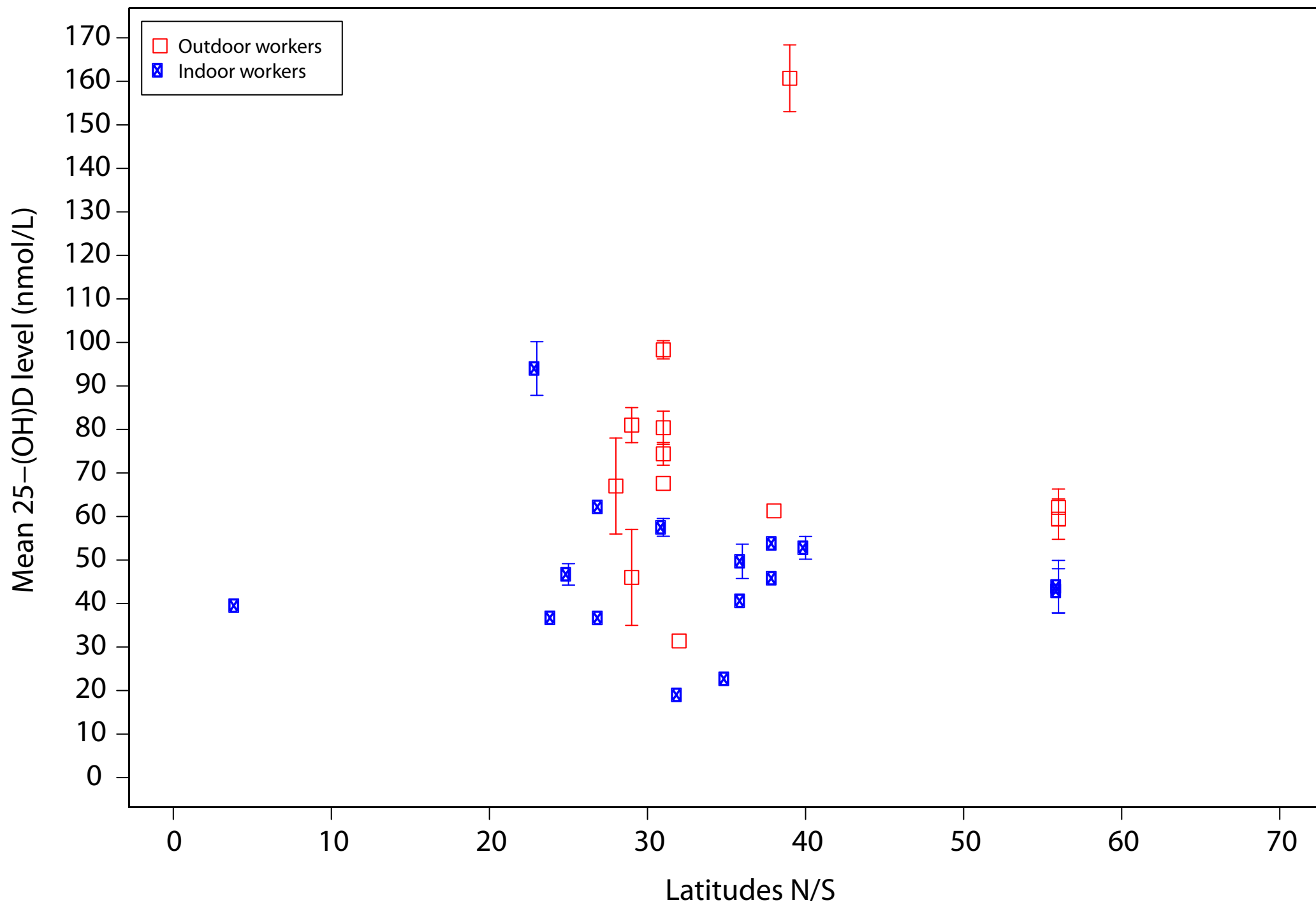

Supplement: Supplementary file 3 — 25-(OH)D levels. Figure S4A. Effect of latitude on serum 25-(OH)D levels in all occupational groups examined. Each data point represents mean ± standard error of the mean of each included study. Inset: symbols and colors of each occupational subgroup. N/S: Northern/Southern hemisphere. Figure S4B. Comparison of serum 25-(OH)D levels in indoor and outdoor workers according to latitude. Each data point represents mean ± standard error of the mean of studies of outdoor and indoor workers included in the analysis. Inset: symbols and colors of each occupational subgroup. N/S: Northern/Southern hemisphere. Figure S4C. Comparison of serum 25-(OH)D levels in healthcare workers according to latitude. Each data point represents mean ± standard error of the mean of studies of each healthcare workers included in the analysis. Inset: symbols and colors of each occupational subgroup. N/S: Northern/Southern hemisphere. (ZIP 229 kb) [file 12889_2017_4436_MOESM3_ESM.zip › Suppl Fig. 4B Latitude and 25-(OH)D outdoor indoorR3.pdf]

Supplementary Fig. 4C

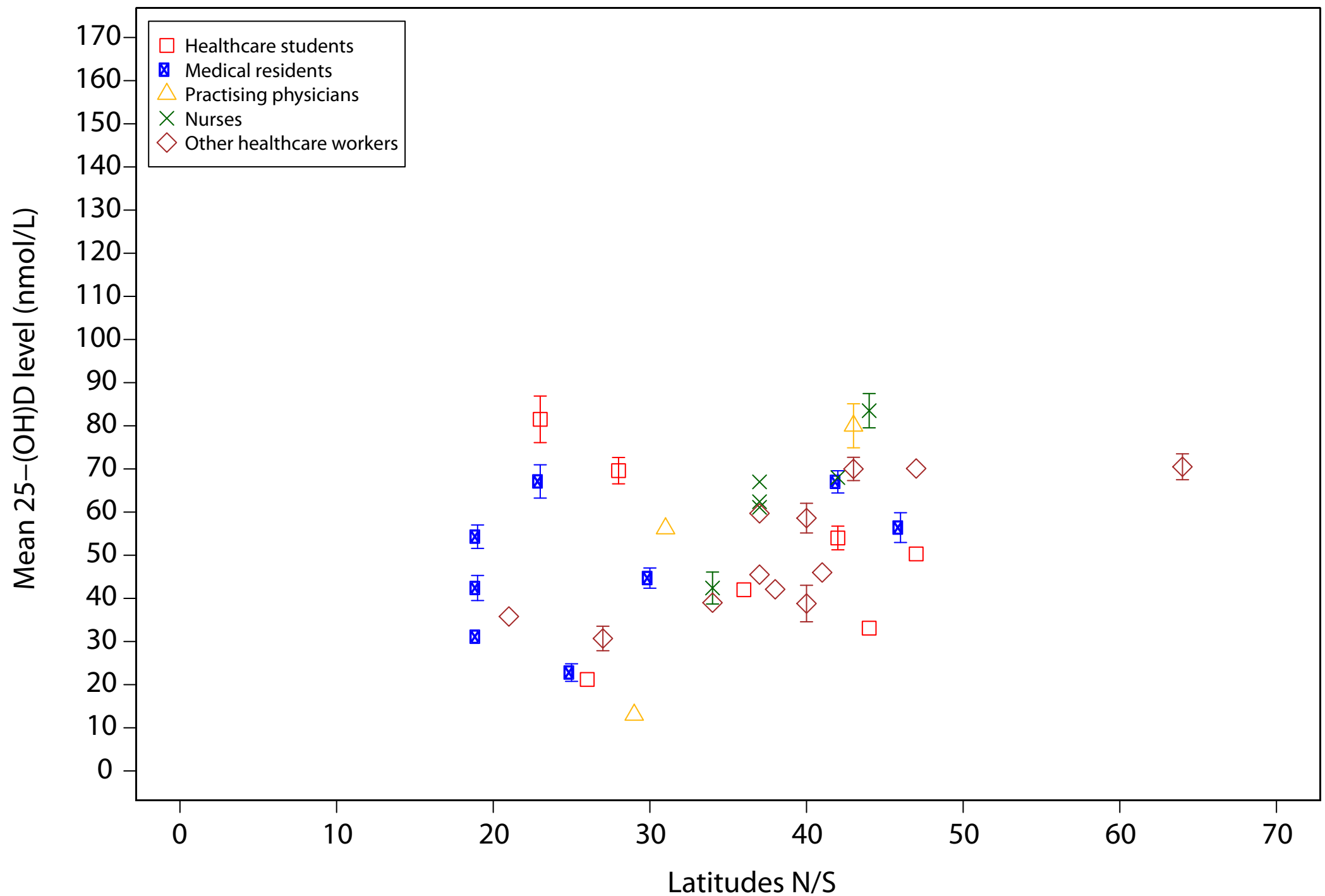

Supplement: Supplementary file 3 — 25-(OH)D levels. Figure S4A. Effect of latitude on serum 25-(OH)D levels in all occupational groups examined. Each data point represents mean ± standard error of the mean of each included study. Inset: symbols and colors of each occupational subgroup. N/S: Northern/Southern hemisphere. Figure S4B. Comparison of serum 25-(OH)D levels in indoor and outdoor workers according to latitude. Each data point represents mean ± standard error of the mean of studies of outdoor and indoor workers included in the analysis. Inset: symbols and colors of each occupational subgroup. N/S: Northern/Southern hemisphere. Figure S4C. Comparison of serum 25-(OH)D levels in healthcare workers according to latitude. Each data point represents mean ± standard error of the mean of studies of each healthcare workers included in the analysis. Inset: symbols and colors of each occupational subgroup. N/S: Northern/Southern hemisphere. (ZIP 229 kb) [file 12889_2017_4436_MOESM3_ESM.zip › Suppl Fig. 4C Latitude and 25-(OH)D healthcareR3.pdf]

Supplementary Fig. 1

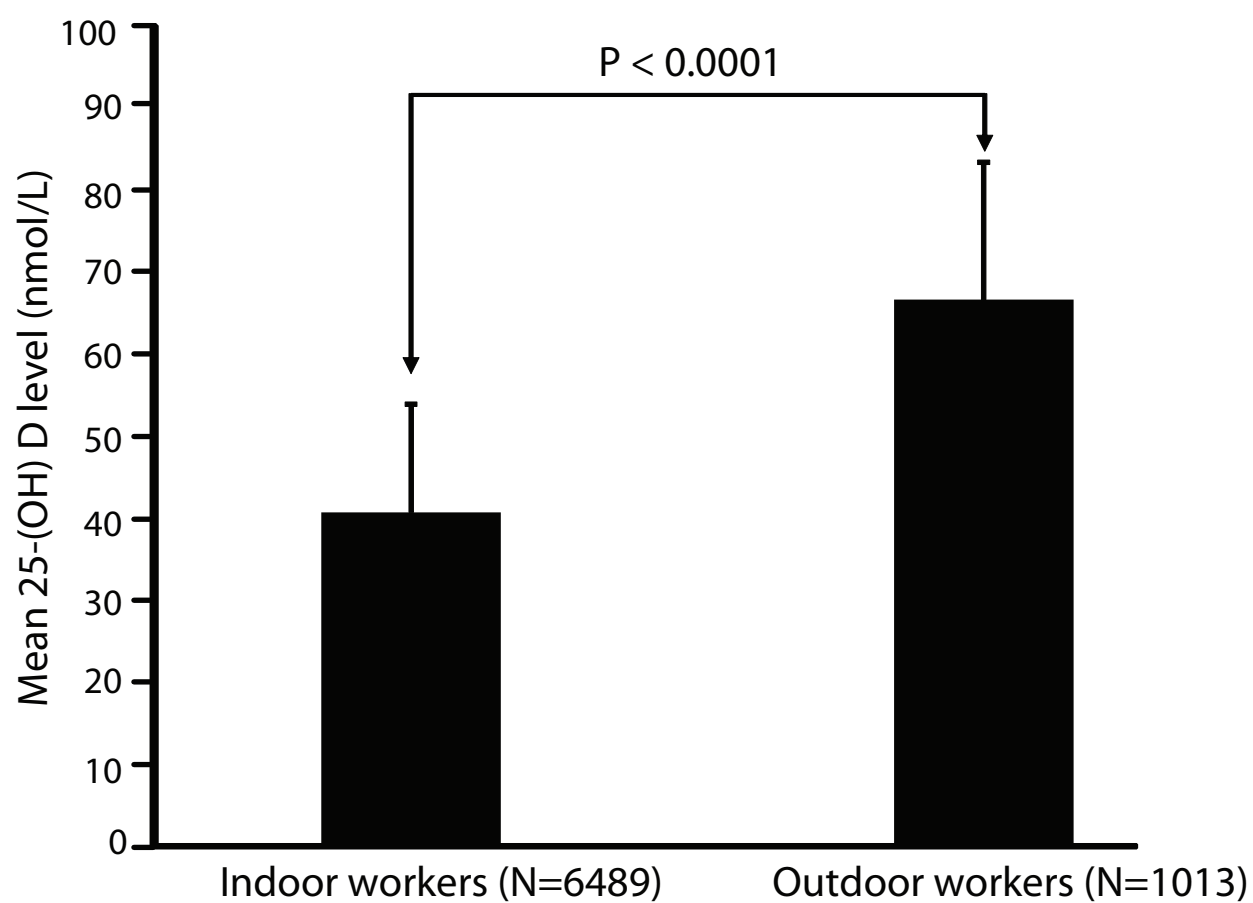

Supplement: Supplementary file 4 — 25-(OH)D levels in indoor and outdoor workers. Figure S1. 25-(OH)D levels in indoor and outdoor workers. Data represent pooled weighted mean ± pooled standard error of the mean for each group. * Statistically significant compared with indoor workers (p < 0.0001). (PDF 212 kb) [file 12889_2017_4436_MOESM4_ESM.pdf]

Supplementary Fig. 5A

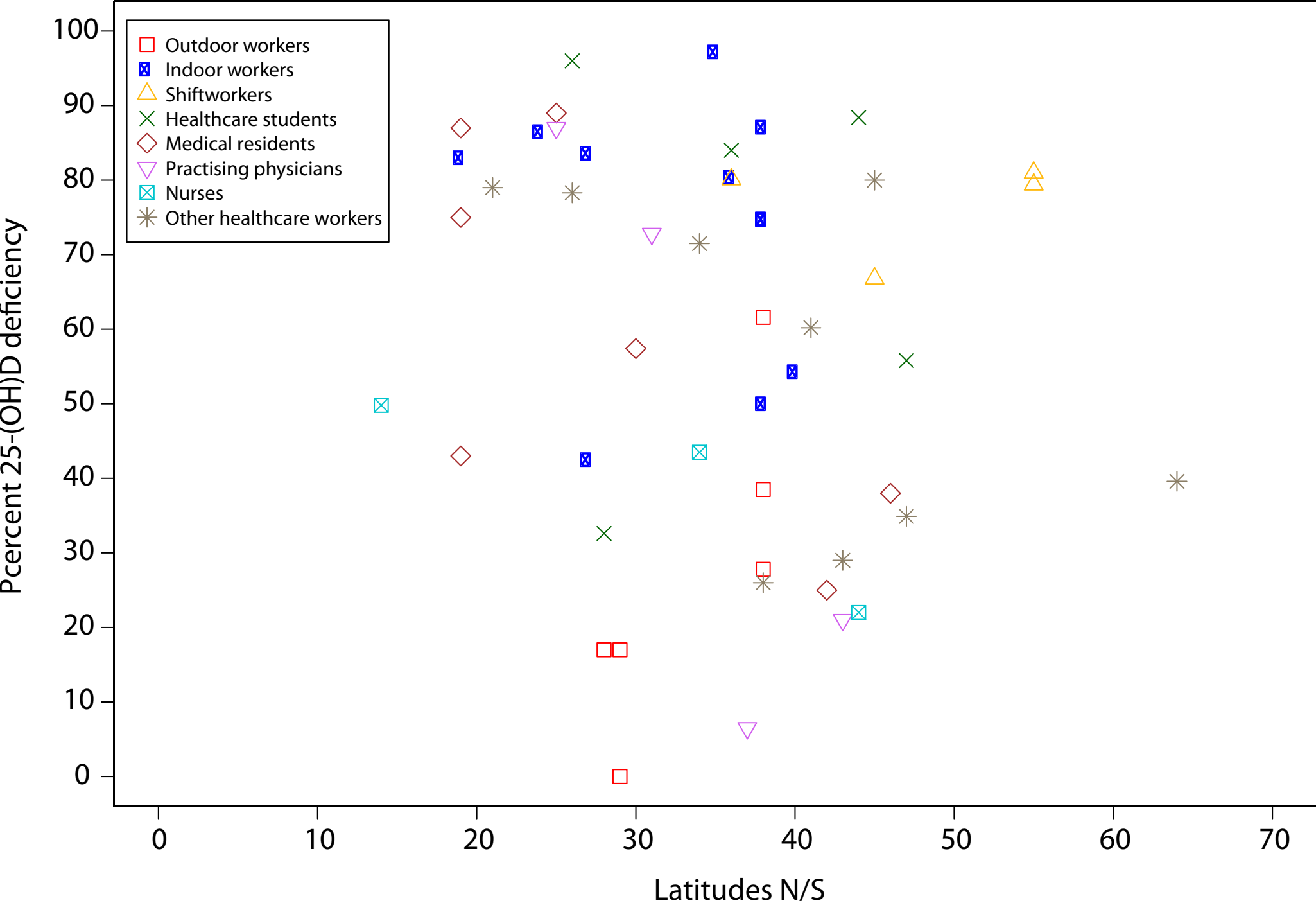

Supplement: Supplementary file 5 — Latitude and % vitamin D deficiency. Figure S5A. Effect of latitude on % vitamin D deficiency in all the occupational groups included in analysis. Percent vitamin D deficiency was defined as the number of subjects of a particular study with mean 25-(OH)D levels less than 50 nmol/L. Inset: symbols and colors of each occupational subgroup. N/S: Northern/Southern hemisphere. Figure S5B. Effect of latitude on % vitamin D deficiency in indoor and outdoor workers included in the analysis. Inset: symbols and colors of each occupational subgroup. N/S: Northern/Southern hemisphere. Figure S5C. Effect of latitude on % vitamin D deficiency in healthcare employees included in the analysis. Inset: symbols and colors of each occupational subgroup. N/S: Northern/Southern hemisphere. (ZIP 195 kb) [file 12889_2017_4436_MOESM5_ESM.zip › Suppl Fig. 5A Latitude and % vitamin D deficiencyR3.pdf]

Supplementary Fig. 5B

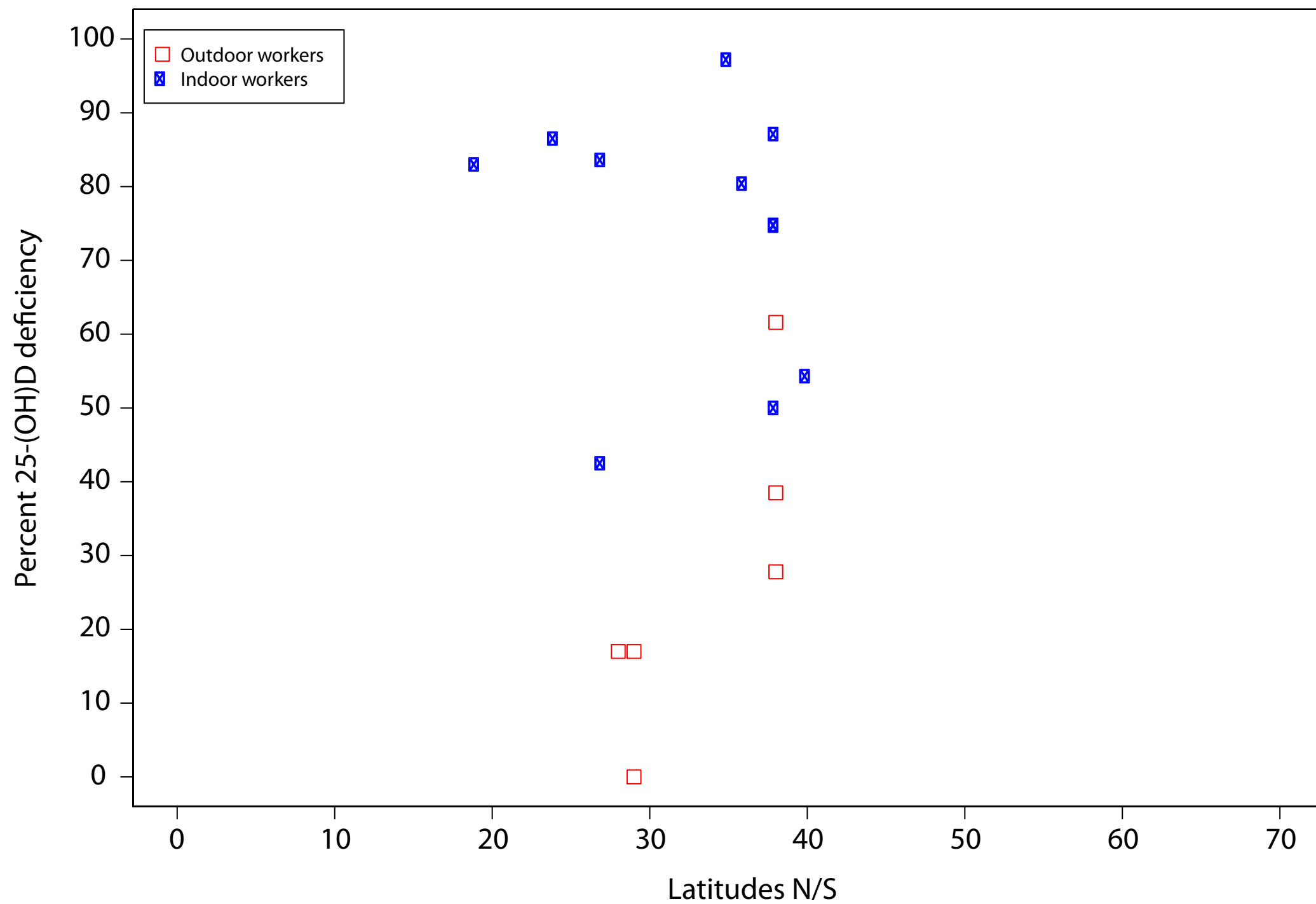

Supplement: Supplementary file 5 — Latitude and % vitamin D deficiency. Figure S5A. Effect of latitude on % vitamin D deficiency in all the occupational groups included in analysis. Percent vitamin D deficiency was defined as the number of subjects of a particular study with mean 25-(OH)D levels less than 50 nmol/L. Inset: symbols and colors of each occupational subgroup. N/S: Northern/Southern hemisphere. Figure S5B. Effect of latitude on % vitamin D deficiency in indoor and outdoor workers included in the analysis. Inset: symbols and colors of each occupational subgroup. N/S: Northern/Southern hemisphere. Figure S5C. Effect of latitude on % vitamin D deficiency in healthcare employees included in the analysis. Inset: symbols and colors of each occupational subgroup. N/S: Northern/Southern hemisphere. (ZIP 195 kb) [file 12889_2017_4436_MOESM5_ESM.zip › Suppl Fig. 5B Latitude and % vitamin D def out inR3.pdf]

Supplementary Fig. 5C

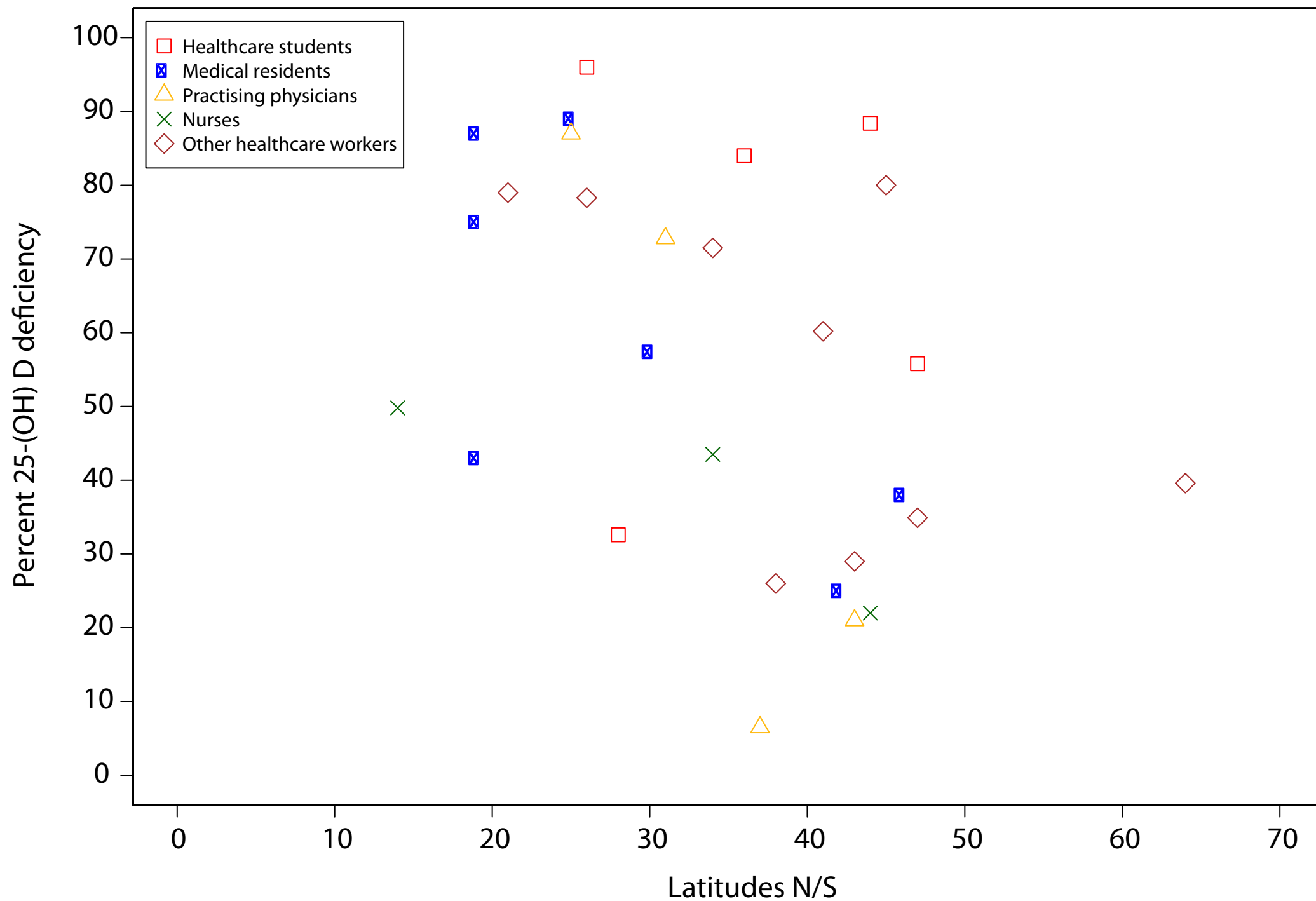

Supplement: Supplementary file 5 — Latitude and % vitamin D deficiency. Figure S5A. Effect of latitude on % vitamin D deficiency in all the occupational groups included in analysis. Percent vitamin D deficiency was defined as the number of subjects of a particular study with mean 25-(OH)D levels less than 50 nmol/L. Inset: symbols and colors of each occupational subgroup. N/S: Northern/Southern hemisphere. Figure S5B. Effect of latitude on % vitamin D deficiency in indoor and outdoor workers included in the analysis. Inset: symbols and colors of each occupational subgroup. N/S: Northern/Southern hemisphere. Figure S5C. Effect of latitude on % vitamin D deficiency in healthcare employees included in the analysis. Inset: symbols and colors of each occupational subgroup. N/S: Northern/Southern hemisphere. (ZIP 195 kb) [file 12889_2017_4436_MOESM5_ESM.zip › Suppl Fig. 5C Latitude and % vitamin D def healthcareR3.pdf]

Supplementary Fig. 6A

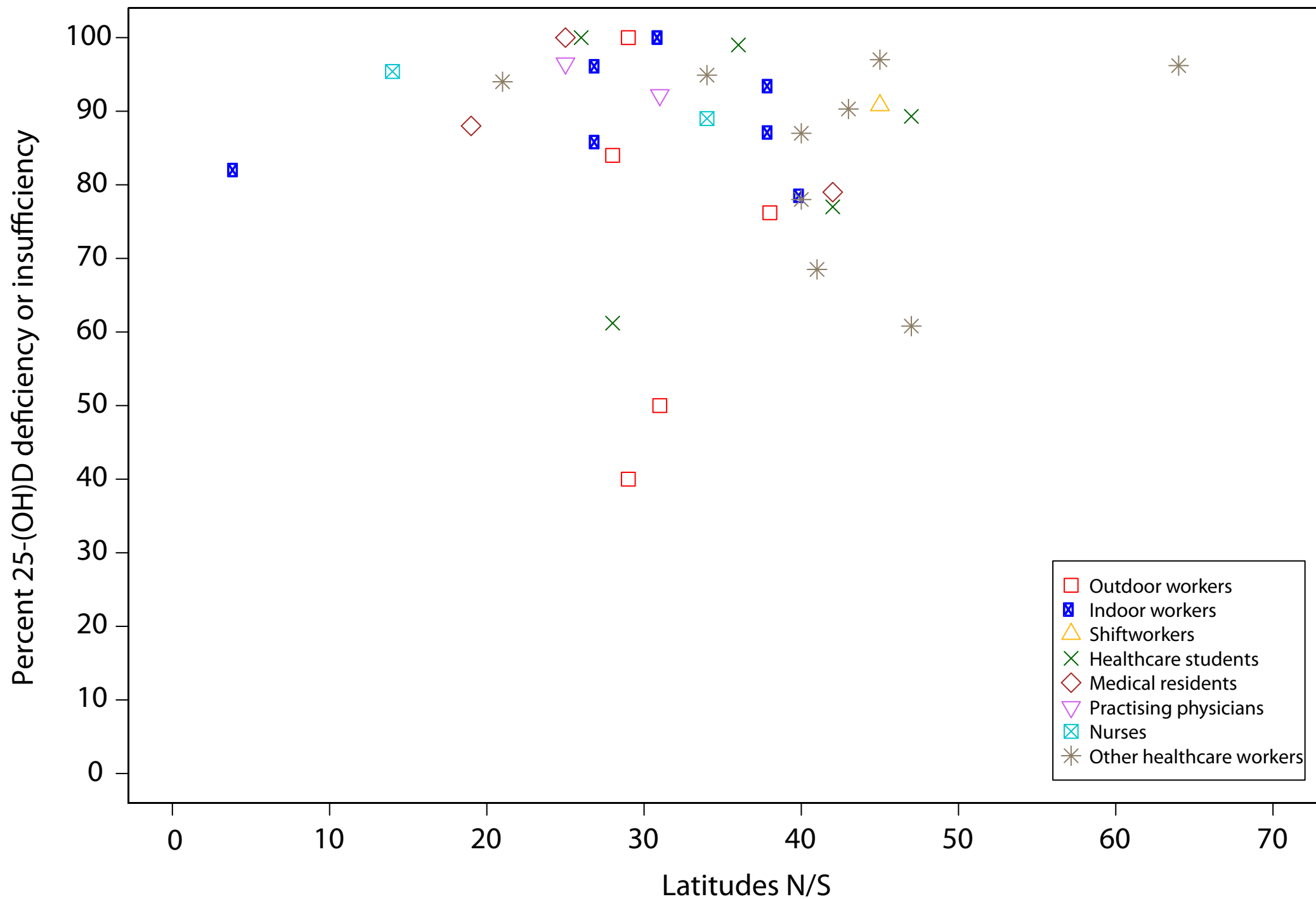

Supplement: Supplementary file 6 — Latitude and % vitamin D deficiency or insufficiency. Figure S6A. Effect of latitude on % vitamin D deficiency or insufficiency in all occupational groups examined. Percent vitamin D insufficiency was defined as the number of subjects of a particular study with mean 25-(OH)D levels less than 75 nmol/L. Inset: symbols and colors of each occupational group. N/S: Northern/Southern hemisphere. Figure S6B. Effect of latitude on % vitamin D deficiency or insufficiency in indoor and outdoor workers. Inset: symbols and colors of each occupational subgroup. N/S: Northern/Southern hemisphere. Figure S6C. Effect of latitude on % vitamin D deficiency or insufficiency in healthcare professionals. Inset: symbols and colors of each occupational subgroup. N/S: Northern/Southern hemisphere. (ZIP 189 kb) [file 12889_2017_4436_MOESM6_ESM.zip › Suppl Fig. 6A Latitude and % vitamin D def or insuffR3.pdf]

Supplementary Fig. 6B

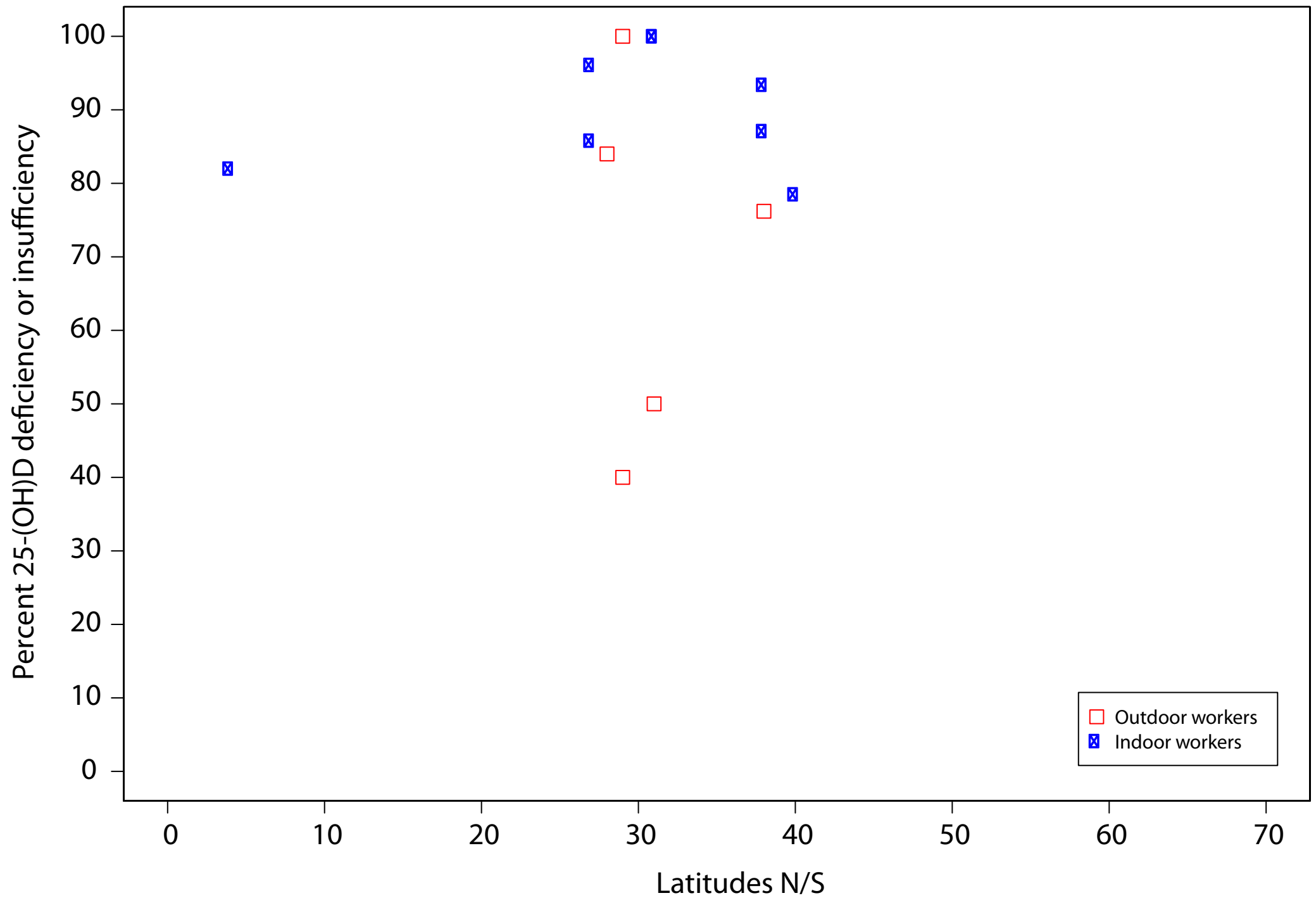

Supplement: Supplementary file 6 — Latitude and % vitamin D deficiency or insufficiency. Figure S6A. Effect of latitude on % vitamin D deficiency or insufficiency in all occupational groups examined. Percent vitamin D insufficiency was defined as the number of subjects of a particular study with mean 25-(OH)D levels less than 75 nmol/L. Inset: symbols and colors of each occupational group. N/S: Northern/Southern hemisphere. Figure S6B. Effect of latitude on % vitamin D deficiency or insufficiency in indoor and outdoor workers. Inset: symbols and colors of each occupational subgroup. N/S: Northern/Southern hemisphere. Figure S6C. Effect of latitude on % vitamin D deficiency or insufficiency in healthcare professionals. Inset: symbols and colors of each occupational subgroup. N/S: Northern/Southern hemisphere. (ZIP 189 kb) [file 12889_2017_4436_MOESM6_ESM.zip › Suppl Fig. 6B Latitude and % vitamin D def insuff out inR3.pdf]

Supplementary Fig. 6C

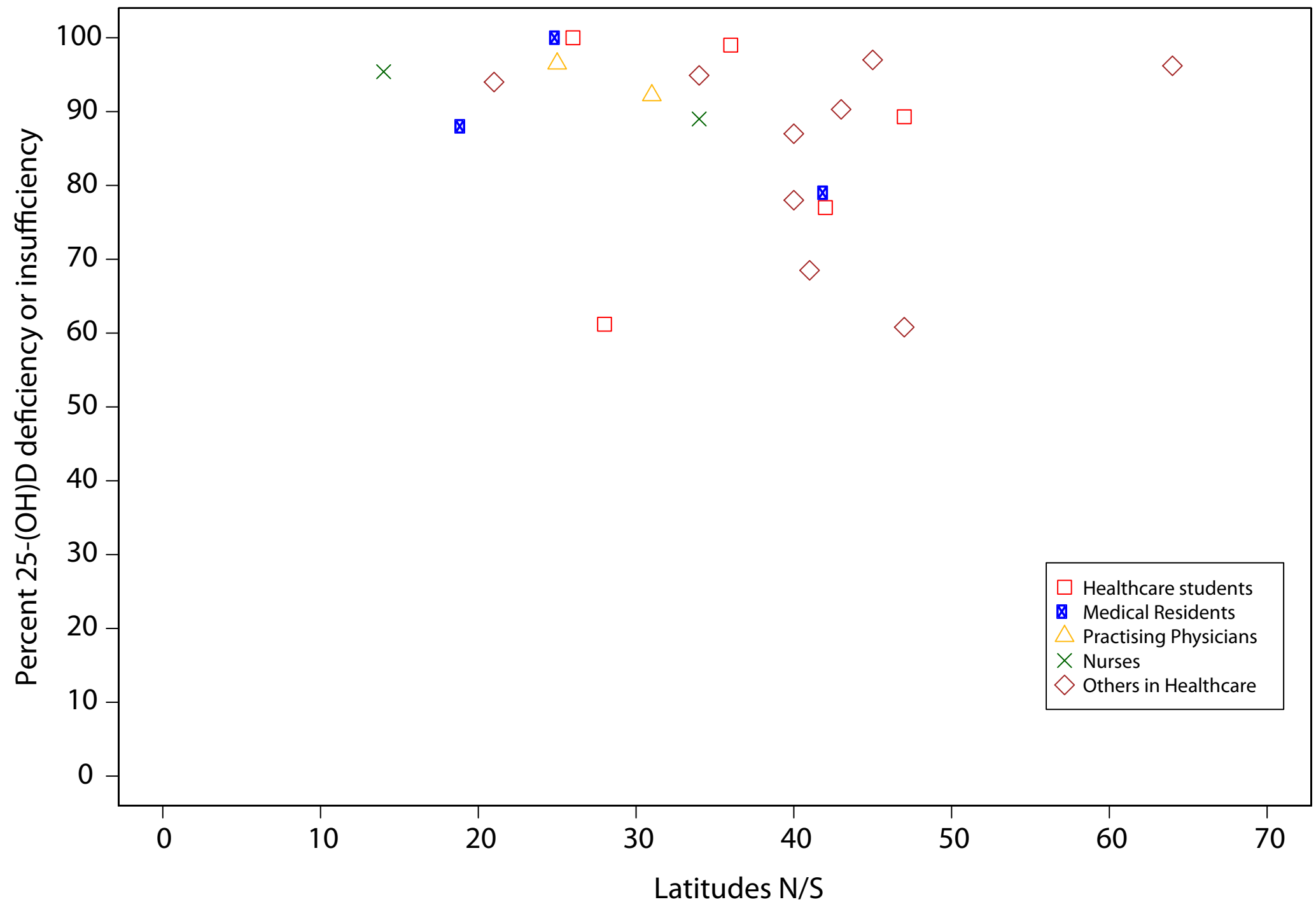

Supplement: Supplementary file 6 — Latitude and % vitamin D deficiency or insufficiency. Figure S6A. Effect of latitude on % vitamin D deficiency or insufficiency in all occupational groups examined. Percent vitamin D insufficiency was defined as the number of subjects of a particular study with mean 25-(OH)D levels less than 75 nmol/L. Inset: symbols and colors of each occupational group. N/S: Northern/Southern hemisphere. Figure S6B. Effect of latitude on % vitamin D deficiency or insufficiency in indoor and outdoor workers. Inset: symbols and colors of each occupational subgroup. N/S: Northern/Southern hemisphere. Figure S6C. Effect of latitude on % vitamin D deficiency or insufficiency in healthcare professionals. Inset: symbols and colors of each occupational subgroup. N/S: Northern/Southern hemisphere. (ZIP 189 kb) [file 12889_2017_4436_MOESM6_ESM.zip › Suppl Fig. 6C Latitude and % vitamin D def insuff healthcareR3.pdf]

Supplementary Fig. 2

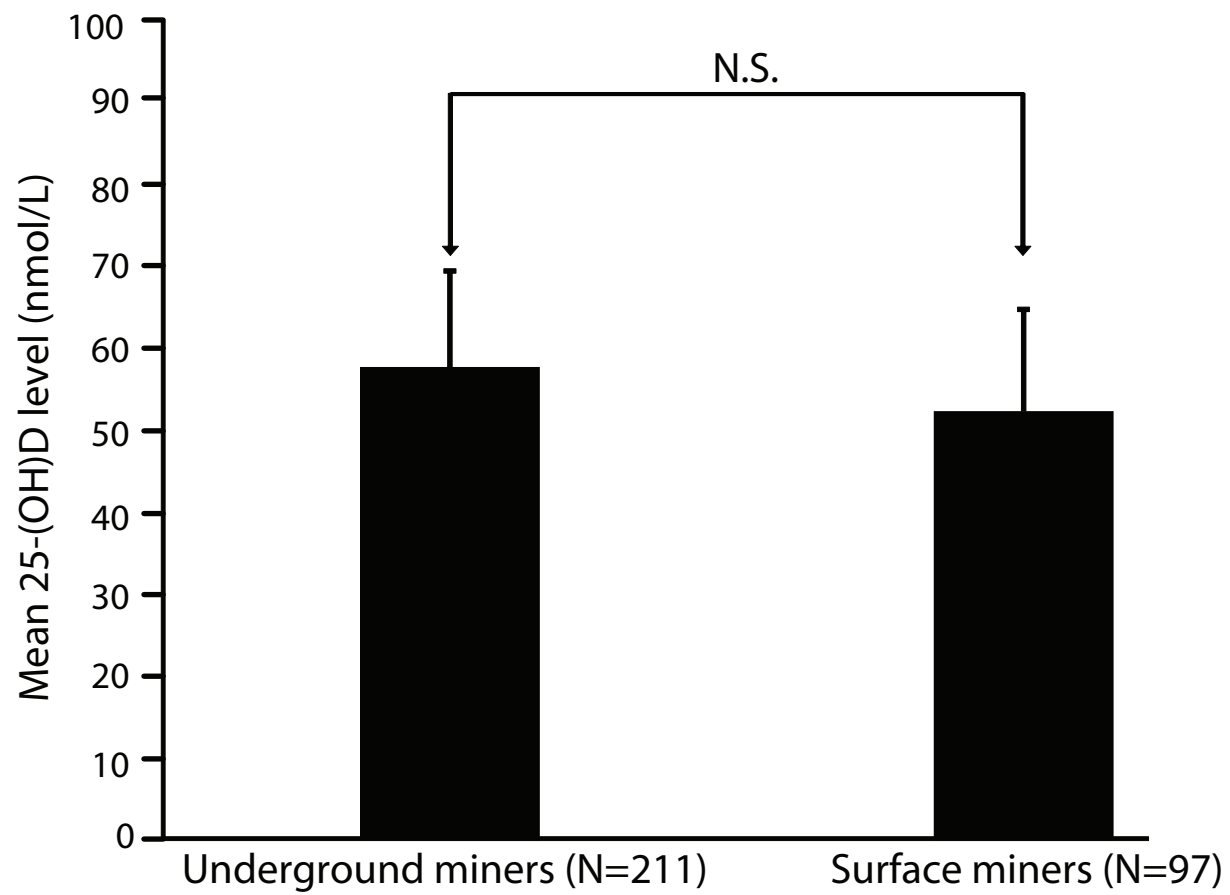

Supplement: Supplementary file 7 — 25-(OH)D levels in underground and surface coalminers. Figure S2. 25-(OH)D levels in underground and surface coalminers. Data represent pooled weighted mean ± pooled standard error of the mean for each group. NS, no significant difference. (PDF 213 kb) [file 12889_2017_4436_MOESM7_ESM.pdf]

Supplementary Fig. 3

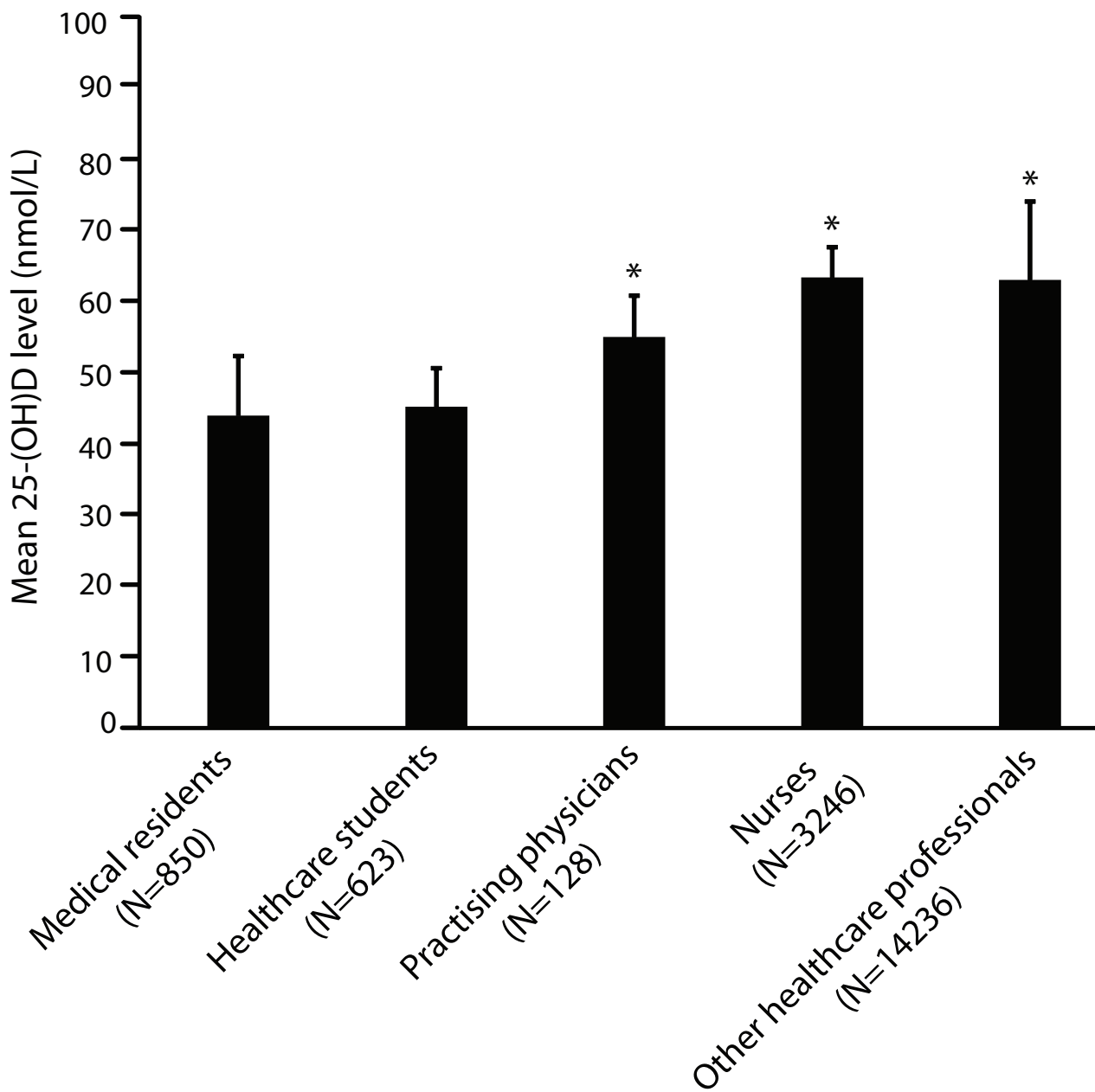

Supplement: Supplementary file 8 — 25-(OH)D levels in healthcare professionals. Figure S3. 25-(OH)D levels among different healthcare professionals. Data represent pooled weighted mean ± pooled standard error of the mean for each healthcare category. * Statistically significant compared to medical residents (p < 0.05). (PDF 222 kb) [file 12889_2017_4436_MOESM8_ESM.pdf]
